# Physical Activity and Adolescent Brain Structure, Mental Health, and Academic Outcomes

**DOI:** 10.1001/jamanetworkopen.2026.36973

**Published:** 2026-10-01

**Authors:** Qingyang Li, Amanda Paluch, Divyangana Rakesh

**Affiliations:** 1 Department of Neuroimaging, Institute of Psychiatry, Psychology & Neuroscience, King’s College London, London, United Kingdom; 2 Department of Kinesiology, University of Massachusetts Amherst; 3 Institute for Applied Life Sciences, University of Massachusetts Amherst

## Abstract

**Importance:**

Urbanization and digitalization are contributing to reductions in the physical activity of adolescent individuals. Daily step count is an increasingly accessible and widely used metric for quantifying physical activity, yet little is known about how it is associated with mental health, academic performance, and brain health in adolescents.

**Objective:**

To investigate the association of daily step count and future mental health and academic performance in adolescents, and whether differences in brain structure mediate these associations.

**Design, Setting, and Participants:**

This cohort study used data from participants in the Adolescent Brain Cognitive Development (ABCD) Study (release 5.1), a large-scale, longitudinal investigation designed to comprehensively characterize psychological and neurobiological development from late childhood to adolescence. The ABCD Study was conducted across 21 sites across the US, providing a diverse and geographically distributed sample. Participants were adolescent individuals with valid daily step count data and usable neuroimaging data collected between November 2018 to November 2020. Data were analyzed from May 2025 to August 2026.

**Exposure:**

Pedometer-based measurements of average daily step count over the previous 3 weeks.

**Main Outcomes and Measures:**

Linear mixed models examined associations of daily step count with mental health, academic performance, and brain structure, and assessed how these associations varied between different step count categories. Using structural equation modeling, step-count–associated brain morphology was examined to determine whether it statistically mediated the association between physical activity and mental health, and/or academic outcomes 1 year later. Dose-response associations were also examined.

**Results:**

This study included 5515 adolescent participants (2640 [47.9%] female; mean [SD] age 11.93 [0.65] years) using step counters. Higher step count was associated with lower total mental health problems (B [SE], −0.080 [0.015]; *t* = −5.497; *P* < .001) and greater cortical thickness in the paracentral (B [SE], 0.057 [0.016]; *t* = 3.621; *P* < .001) and rostral anterior cingulate (B[SE], 0.052 [0.016]; *t* = 3.319; *P* = .001) regions. Further analyses showed a significant dose-response association, with progressively lower mental health problems across higher step categories compared with the under 5000-step group. Mediation analyses revealed that greater paracentral thickness statistically mediated the association between higher daily step count and fewer mental health problems 1 year later.

**Conclusions and Relevance:**

In this cohort study, higher daily step counts were associated with structural differences in the brains of adolescents, which were in turn associated with better mental outcomes prospectively. Beneficial associations for mental health appeared to accrue continuously across the observed range, with no evidence of a threshold or plateau, highlighting that every step counts and the potential value of promoting daily steps in adolescents.

## Key Points

**Question:**

Is daily step count associated with mental health, academic performance, and brain structure in early adolescents?

**Findings:**

In this cohort study of 5515 adolescent participants (aged 12 years) outfitted with step counters, higher daily step count was associated with better mental health in a dose-response manner. Greater cortical thickness statistically mediated the association of higher daily step count and better mental health 1 year later.

**Meaning:**

These findings suggest that accumulating daily steps represents an accessible target associated with positive mental health outcomes and brain development in adolescents.

## Teaser

This cohort study investigates associations between daily step count and future mental health and academic performance in adolescents.

## Introduction

Higher levels of physical activity are associated with lower incidence of mental health problems as well as higher cognitive functioning and academic performance in youths.^1,2,3,4,5^ Early adolescence, marking the transition from childhood to adolescence, may represent a key developmental window for experience-dependent plasticity,^6,7,8^ during which physical activity may shape brain maturation, with potential long-term benefits on social and cognitive outcomes.^9^ However, the neurobiological mechanisms underlying the associations between physical activity and positive outcomes remain poorly understood. It is estimated that approximately 81% of adolescents globally are not reaching the current World Health Organization recommendation of being active 60 minutes per day 5 days per week^10^; research on the associations of physical activity with brain structure to support mental health and academic outcomes in adolescents is crucial. Such research can inform interventions aimed at promoting adolescent well-being.

Emerging evidence suggests an association between daily physical activity and brain structure in adolescents. For example, higher self-reported 24-hour and past-week physical activity was associated with higher cortical thickness and surface area in the caudal middle frontal gyrus, rostral anterior cingulate,^11,12^ medial pericalcarine, cuneus, and precuneus.^13^ Accelerometer data further revealed 24-hour activity to be correlated with vertex-wise shape expansions in the subcortex, including in the pallidum, putamen, and thalamus.^14^ Several cross-sectional studies and randomized clinical trials have also reported associations between physical activity and hippocampal volume,^15,16,17^ whereas other studies, including a meta-analysis across the lifespan, found no significant association between exercise and total hippocampal volume.^18,19^ Together, these findings highlight the potential role of physical activity in shaping both cortical and subcortical brain structure during adolescence.

However, existing studies associating physical activity with adolescent brain structure have faced several limitations. First, prior work relied largely on subjective self-reports or accelerometer-based data from brief windows (eg, 24 hours) which are susceptible to reporting bias and recall error and/or do not represent habitual activity patterns. Daily step count assessed through wearable technology has emerged as a simple, practical, and scalable metric of physical activity levels^20,21^ and is known to be associated with health outcomes in young people.^22^

Step counts offer a population-friendly alternative to intensity-based metrics that show moderate tracking from childhood to adolescence,^23^ and therefore serve as a practical proxy for habitual physical activity. However, no studies have examined how step counts relate to adolescent mental health, academic performance, and brain development, which could inform practical and cost-effective interventions. Additionally, prior work is limited by small samples (approximately 100 participants) and low sociodemographic diversity, leading to reduced statistical power and lower generalizability.

Moreover, previous studies have primarily adjusted for household socioeconomic status (SES), often overlooking the confounding role of neighborhood variables (eg, neighborhood disadvantage and green space access), which are associated with physical activity, brain structure, mental health, and academic performance.^24,25,26,27,28,29^ Most importantly, despite clear evidence linking higher levels of physical activity to lower mental health problems and higher academic performance,^3,30,31^ and the relevance of brain structure for these outcomes,^32,33,34,35,36^ no studies, to our knowledge, have examined whether brain structural differences mediate these associations.

To address these gaps in the literature, we investigated associations between daily step count, mental health, academic performance, and brain structural outcomes, adjusting for household and neighborhood factors. Additionally, we examined whether brain structure statistically mediates the association between daily step count and future mental health and academic performance. Previous studies have commonly modeled step count as a continuous exposure.^37,38^ To explore whether the associations plateau at higher levels, we also modeled step count categorically. Based on a previous accelerometer-based magnetic resonance imaging finding,^14^ we hypothesized that higher step count would correlate with larger pallidum, putamen, and thalamus volumes. Given that cortical thickness and surface area have distinct developmental trajectories^39,40^ and show different patterns of associations with both physical activity and fitness measures,^13,41^ we examined these measures separately from cortical volume. Based on existing cross-sectional evidence on these measures,^11,12,13^ we hypothesized greater cortical thickness and surface area in frontal and parietal regions. We further hypothesized that these brain measures would mediate the associations between higher step count and better mental health and academic outcomes.

## Methods

In this cohort study, participants were drawn from the Adolescent Brain Cognitive Development (ABCD) Study (release 5.1). The ABCD Study recruited 11 879 children (9-10 years of age) from 21 sites across the US to comprehensively characterize psychological and neurobiological development during adolescence.^42^ Approval was received from the University of California, San Diego, which served as the central institutional review board, with reliance agreements established across all 21 data collection sites. All parents submitted their written, informed consent, and all children gave their consent. Analysis plans were preregistered on the Open Science Framework.^43^ The study followed the Strengthening the Reporting of Observational Studies in Epidemiology (STROBE) reporting guidelines.

### Measures

#### Daily Step Count

Participants wore Fitbit Charge 2 devices on the wrist for 3 weeks to monitor daily step counts before the 2-year follow-up assessment, when participants were approximately 11 to 12 years old, the earliest time point with available step-count data. Additionally, previous research has shown that pedometer-assessed step counts have substantial explanatory power for moderate-to-vigorous physical activity in children.^37^ The validity of the step counter used has been supported in previous studies^44,45^ (see eMethods in Supplement 1). ABCD Study protocols required at least 600 minutes per day of valid recordings for at least 4 weekdays and at least 1 weekend day per week.^45^ The mean daily step count was calculated by averaging all valid daily step counts across the 3-week period. Histograms and correlations among the study variables are presented in eFigure 1 and 2 in Supplement 1.

#### Mental Health and Academic Performance

Participant-reported total mental health problems at the 3-year follow-up were assessed using the Brief Problem Monitor, a 19-item measure of internalizing, externalizing, and attention symptoms.^46^ The monitor is an abbreviated version of the Child Behavior Checklist and has demonstrated good psychometric validity^47^ (eMethods in Supplement 1). Academic performance at the 3-year follow-up was measured using the average of parent- and youth-reported school grades, rated on a scale from 1 (an A+ grade) to 12 (failing). Scores were reverse-coded so that higher values indicate better performance.

#### Covariates

Consistent with previous studies,^11,13,14^ household and neighborhood SES, access to green spaces, and body mass index (BMI, calculated as weight in kilograms divided by height in meters squared) were included as covariates as they are associated with both physical activity and the study outcomes, including brain structure, mental health, and academic performance.^24,48,49,50,51,52,53,54,55,56^ Household SES was assessed using the income-to-needs ratio (INR), which accounts for household size relative to the federal poverty threshold and parent educational attainment. Neighborhood SES was measured using the Area Deprivation Index (ADI) at the census tract level. Details of its construction have been described in previous studies.^57,58^ Residential greenspace within a 1 kilometer radius was estimated via the satellite-based normalized difference vegetation index. ADI and normalized difference vegetation index were assessed at baseline since associated data were available only at baseline, while INR and parental education were assessed at the 2-year follow-up. BMI was assessed at the 1-year follow-up to avoid potential bidirectionality with step count^59,60^ (eMethods and eFigure 3 in Supplement 1).

### Neuroimaging

T1-weighted images were obtained using standardized protocols on 3T magnetic resonance imaging scanners from Siemens, Philips, or General Electric, each equipped with a 32-channel head coil.^61,62^ Image preprocessing was carried out by the ABCD Data Analysis, Informatics, and Resource Center.^62,63^ Cortical surface reconstruction and parcellation of cortical and subcortical structures were performed using FreeSurfer version 7.1.1 (Laboratory for Computational Neuroimaging) with the Desikan-Killiany atlas. We examined thickness and surface area of 34 cortical regions, volume of 7 subcortical regions, and 3 whole-brain measures, including total surface area, average cortical thickness, and total cortical volume. Because there were no estimates of lateralized effects, values across hemispheres were averaged for analyses. This resulted in 78 dependent variables. Scans were excluded based on quality control criteria specified by the ABCD Data Analysis, Informatics, and Resource Center.^62^

### Statistical Analyses

Statistical analyses comprised primary linear mixed-effects models examining associations between daily step count and mental health, academic performance, and brain structure measures, followed by mediation analyses and exploratory and/or sensitivity analyses. We first examined the association between daily step count and mental health and academic performance 1 year later, as well as brain structure, using linear mixed effects models (LMM) in the lmer package in R version 4.5.2 (R Project for Statistical Computing)^64^ with daily step count as the independent variable and (1) mental health, (2) academic performance, and (3) each brain measure as the dependent variable in separate models. *P* values were estimated using Satterthwaite approximation. Given the limited evidence in this area, we conducted exploratory whole-brain analyses beyond our a priori hypotheses. Specifically, we examined associations with both global brain structure metrics (ie, average thickness, total surface area, and total volume) as well as regional thickness and surface area and subcortical volume. To examine potential curvilinear associations, we added a quadratic term for step count to the original models. We then compared models with and without the quadratic term using likelihood ratio tests, with the better-fitting model retained for primary analyses.

To investigate whether brain structure statistically mediates the association of daily step count with future mental health and academic performance, we used structural equation modeling (Lavaan package in R version 4.5.2 [R Project for Statistical Computing]).^65^ As preregistered, only the measures that showed significant associations with daily step count in the LMM analyses were included in the mediation analyses. In each model, daily step count was included as the independent variable, the relevant brain structure measure as the mediator, and mental health or academic performance as the outcome variable (in separate models). Continuous variables (including daily step count, brain structural measures, and covariates) were standardized before analyses.

#### Covariates and Multiple Comparison Correction

We controlled for sex (parent-reported), age, INR, ADI, BMI, green space, parents’ highest education, scanner model as fixed effects and included random effects for both family identifier (ID) and acquisition site. In the mediation analysis, as lavaan allows only one clustering variable at a time, and models clustering by site while adjusting for scanner did not converge because of their high collinearity, we included family ID as the single clustering variable. For the brain analyses, we adjusted for the false discovery rate (FDR; *P* < .05; 2-tailed) to correct for multiple comparisons within each set of analyses (ie, cortical thickness, surface area, and subcortical volume; 34, 34, and 7 comparisons, respectively; 3 comparisons for whole-brain measures). For mediation analyses, we applied FDR correction within analyses for each outcome variable (ie, academic performance and mental health).

#### Exploratory Aim: Categorical Analysis

We used the same analytical framework described previously and modeled step count as a categorical variable based on quartiles within a single model. To facilitate interpretation across meaningful activity thresholds, step counts were grouped into 5 categories: (1) under 5000 steps, (2) 5000 to 7499 steps, (3) 7500 to 9999 steps, (4) 10 000 to 11 999 steps, and (5) at least 12 000 steps per day, with under 5000 steps as the reference group. These boundaries align closely with the sample quartiles (7366, 9323, and 11 446 steps), ensuring the categories reflect the observed activity distribution.

#### Sensitivity Analyses

To assess the robustness of the findings, we conducted several sensitivity analyses. For both the LMMs and mediation analyses, we additionally included a medical history score to account for potential health-related confounding,^66^ and replaced raw BMI with age- and sex-standardized BMI *z* scores based on the Centers for Disease Control growth records.^67^ We further restricted analyses to participants with 3 weeks of valid step count data (those meeting the ABCD wear-time criteria). Models of mental health and academic performance, including mediation, were additionally adjusted for prior measures of the corresponding outcome (brief problem monitor at 1-year follow-up and academic performance at the 2-year follow up, which was the first time it was collected). Models of neuroimaging were additionally adjusted for intracranial volume to account for individual differences in head size. For the LMMs only, we conducted propensity-weighted linear regressions using the Survey package in R version 4.5.2 (R Project for Statistical Computing). We used the ABCD Study’s baseline American Community Survey–raked propensity weights, which account for demographic differences between the ABCD sample and the target US population.^68^ As preregistered, we also report LMM estimates with family-level clustering only. For mediation analyses only, we reran the models using acquisition site as the clustering variable instead of family ID and removed scanner fixed effects to examine the robustness of the clustering specification (eMethods in Supplement 1). Sensitivity analyses were considered significant at *P* < .05.

## Results

This study included 5515 adolescent participants (2640 [47.9%] female; mean [SD] age 11.93 [0.65] years) with a mean (SD) daily step count of 9595 (3239) steps per day and a mean daily wear time of 875.55 (66.34) minutes (Table) (eFigure 4 in Supplement 1). Higher step count was associated with lower total mental health problems (B [SE], −0.080 [0.015]; 95% CI, −0.109 to −0.052; *t* = −5.497; *P* < .001; Figure 1C) but not academic performance 1 year later (Figure 1A). Results remained significant when adjusting for prior mental health (eTable 14 in Supplement 1). Models including a quadratic term for step count did not improve model fit (eTables 19 and 20 in Supplement 1).

**Table.** Demographic Information

Characteristic	Participants, mean (SD) (N = 5515)
Sex, No. (%)	
Female	2640 (47.9)
Male	2875 (52.1)
Age, y	11.93 (0.65)
Daily step count^a^	9595 (3239)
Wear time,min/d	875.55 (66.34)
Area Deprivation Index	37.68 (24.67)
Income-to-needs ratio	4.20 (2.39)
Parental education, y	16.14 (2.65)
Body mass index^b^	19.44 (4.29)
Normalized difference vegetation index score^c^	0.46 (0.36)
Brief problem monitor score^d^	0.40 (0.30)
Academic performance score^e^	9.54 (1.94)

^a^ The median (IQR) daily step count was 9323 (7366-11 446) steps per day.

^b^ Body mass index calculated as weight in kilograms divided by height in meters squared.

^c^ Higher scores indicate greater green space exposure.

^d^ Higher scores indicate more mental health problems.

^e^ Higher scores indicate better academic performance.

**Figure 1.**
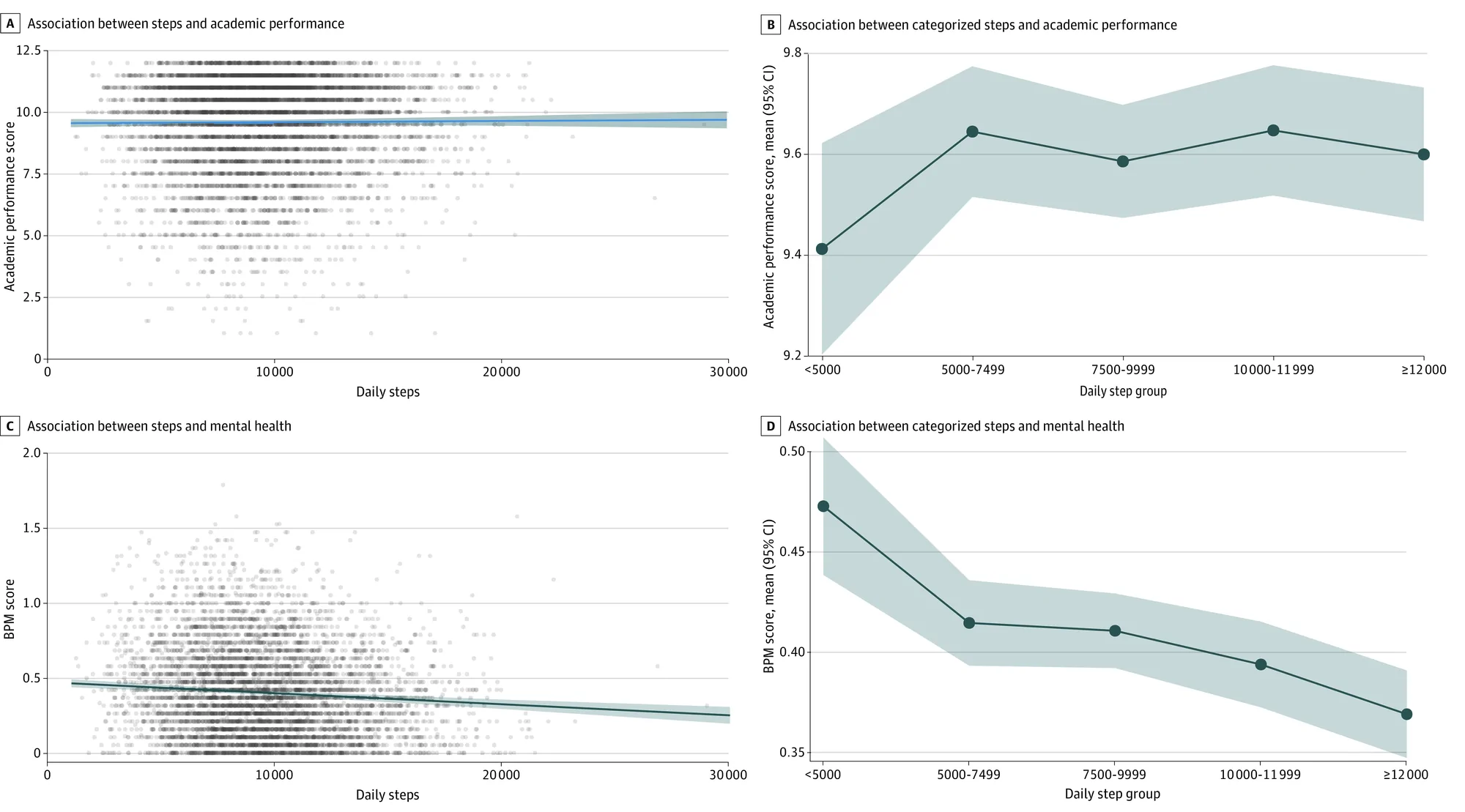
Scatterplots and Line Graphs of Association Between Daily Step Count and Academic Performance and Mental Health Problems 1 Year Later A and C, Dots indicate observed values, lines indicate associations beween daily steps (x-axis) and BPM score (y-axis), and shaded areas indicate 95% CIs. B and D, Shaded area indicates 95% CI. BPM indicates brief problem monitor.

In exploratory analyses, we found that compared with the under 5000-step reference group, adolescents achieving higher step counts had lower incidence of mental health problems. Specifically, the 5000 to 7499 steps per day group, the 7500 to 9999 steps per day group, the 10 000 to 11 999 steps per day group, and greater than 12 000 steps per day group had lower brief problem monitor (BPM) mean scores by 0.048 (95% CI, −0.089 to −0.007; *t* = −2.301; *P* = .02); 0.058 (95% CI, −0.098 to −0.018; *t* = −2.868; *P* = .004), 0.073 (95% CI, −0.115 to −0.032; *t* = −3.452; *P* = .001), and 0.102 points (95% CI, −0.144 to −0.059; *t* = −4.726; *P* < .001; Figure 1D). Pairwise comparisons using Tukey post hoc test showed that the group achieving at least 12 000 steps had lower BPM mean scores than the 5000 to 7499 group and 7500 to 9999 group by 0.053 points (95% CI, 0.014 to 0.092; *P* = .002), and 0.043 points (*P* = .005; 95% CI [0.009 to 0.078]), respectively. This pattern indicates a dose-response gradient: higher step categories were associated with incrementally fewer mental health problems. In addition, compared with the under 5000-step reference group, the 10 000 to 11 999 step group also showed higher academic performance by 0.262 (95% CI, −0.518 to −0.007; *t* = −2.013; *P* = .044; Figure 1B).

### Daily Step Count and Brain Structure

Higher step count was not associated with average cortical thickness, total surface area, or total cortical volume. Models including a quadratic term for step count did not improve model fit (eTable 19 and 20 in Supplement 1). At the regional level, higher step count was associated with greater paracentral (standardized B [SE], 0.057 [0.016]; 95% CI, 0.026 to 0.087; *t* = 3.621; *P* < .001) and rostral anterior cingulate (standardized B [SE], 0.052 [0.016]; 95% CI, [0.021 to 0.082]; *t* = 3.319; *P* = .001) thickness (Figure 2). Models including a quadratic term for step count did not improve model fit (eTable 19 and 20 in Supplement 1). No other findings, including regional surface area or subcortical volume survived FDR correction (eTable 2 in Supplement 1).

**Figure 2.**
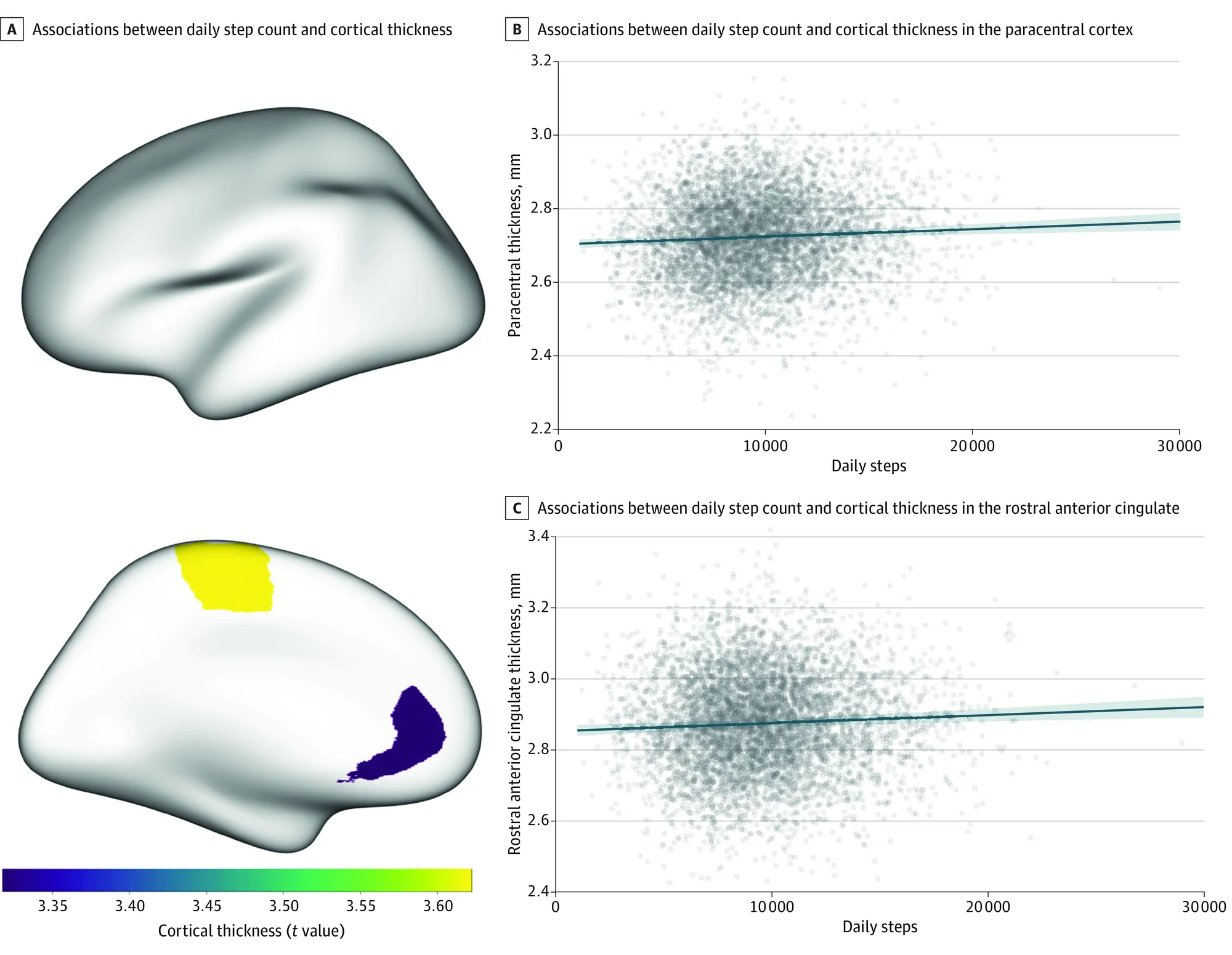
Diagram and Line Graphs of Region-Specific Associations Between Daily Step Count and Cortical Thickness A, Warmer colors reflect higher *t*-stat values and white represents nonsignificant results (eTable 1 in Supplement 1). B and C, Dots indicate observed values, lines indicate associaions between daily steps (x-axis) and brief problem monior score (y-axis), and shaded areas indicate 95% CIs.

In exploratory analyses, compared with the under 5000-step reference group, only the group accumulating at least 12 000 steps (standardized B [SE], 0.142 [0.070]; 95% CI, 0.005 to 0.279; *t* = 2.028; *P* = .004; Figure 3A) demonstrated higher paracentral thickness. No significant differences emerged for rostral anterior cingulate thickness across step categories (Figure 3B). The categorical analyses indicated that the contrast between very low and higher habitual activity was large enough to be detectable at the group level for paracentral thickness. However, the absence of consistent stepwise differences across intermediate categories and the lack of improved fit after including a quadratic term for step count suggest that the association would be better characterized as graded rather than threshold-based.

**Figure 3.**
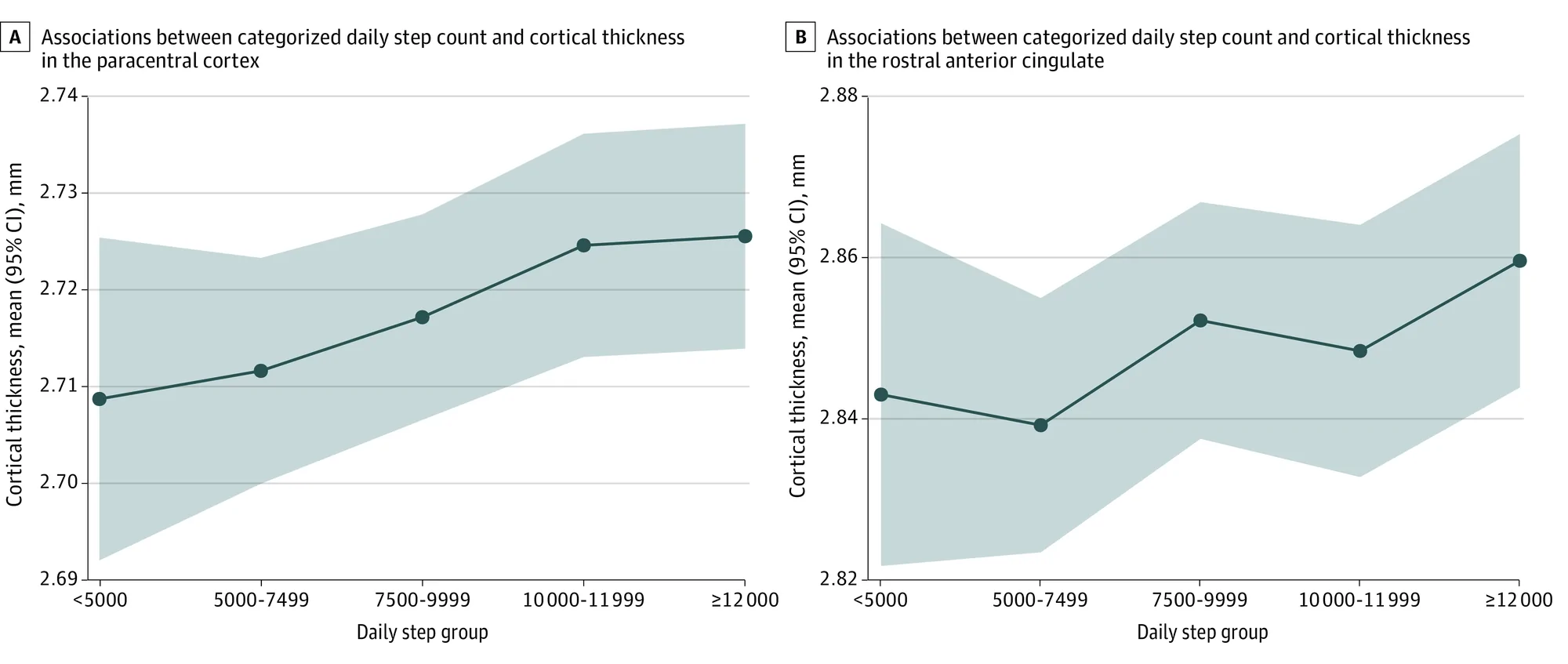
Line Graphs of Associations Between Categorized Daily Step Count and Cortical Thickness in the Paracentral and Rostral Anterior Cingulate Cortices Shaded areas indicate 95% CIs.

### Brain Structure as a Mediator

Paracentral cortical thickness statistically mediated the association of higher daily step count and fewer mental health problems 1 year later (indirect effect B[SE], −0.002 [0.001]; 95% CI, −0.0045 to −0.0004; *P* = .02; Figure 4). Results remained significant after adjusting for prior youth-reported mental health. No other significant indirect associations were observed after FDR correction.

**Figure 4.**
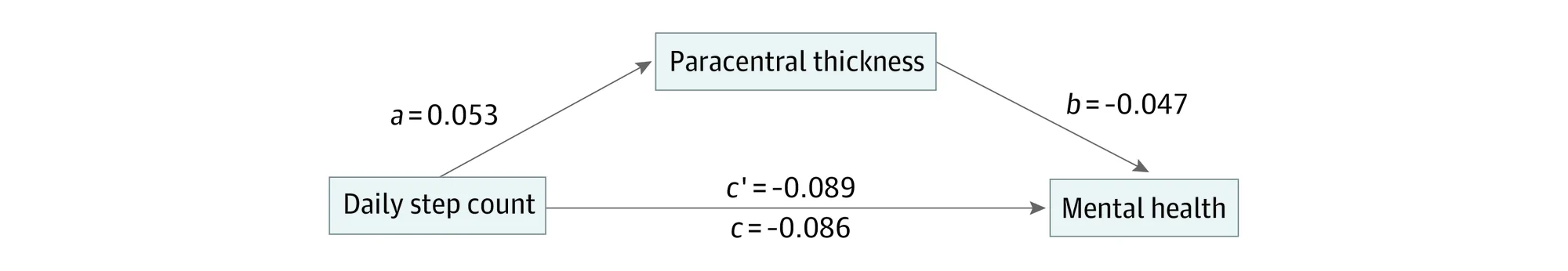
Path Diagram of the Mediation Model Result Complete results, including the not significant results, are provided ineTable 3 in Supplement 1. Path (a) indicates the association between daily steps and brain structure; path (b), the association between brain structure and mental health or academic performance; path (c), the total effect of daily steps on the outcomes; and path (c^1^), the corresponding direct effect.

### Sensitivity Analyses

LMM and mediation results remained consistent after additionally adjusting for BMI *z* score, intracranial volume (in neuroimaging analyses only), and prior medical history. The pattern of results was also unchanged in analyses restricted to participants with complete 3-week step data. Results from mental health and academic performance models remained stable after adjusting for prior BPM and prior academic performance. The LMM results were also robust to propensity weighting and to accounting for family-level clustering only. The mediation results were unchanged when clustering by acquisition site. Complete model output for these analyses have been provided in eTables 4 to 18 in Supplement 1.

## Discussion

In this cohort study, we investigated associations between physical activity—assessed using average daily step count—and brain structure, mental health, and academic performance in a large sample of adolescents. We found that higher daily step count was associated with fewer mental health problems 1 year later. Greater daily step count was also associated with higher cortical thickness in the paracentral and rostral anterior cingulate. Mediation analyses indicated that paracentral thickness statistically mediated the association between higher daily step count and fewer mental health problems 1 year later.

Consistent with prior work on daily physical activity,^2,4,69^ we found that higher daily step count was associated with better mental health prospectively. However, daily steps were not associated with academic performance. Neurophysiological and neurochemical mechanisms are considered to be one pathway through which physical activity may benefit mental health.^30,70^ For example, physical activity stimulates the release of endorphins, dopamine, and serotonin, which may help alleviate stress and promote overall mental well-being.^70^ Physical activity has also been associated with decreased cortisol levels, which may be linked to reduced systemic inflammation through the regulation of pro-inflammatory cytokines,^70,71^ thereby alleviating symptoms of anxiety and depression. Of note, psychological pathways, such as differences in self-regulation, may also underlie this step-mental health association.^30^ In contrast, academic performance is more closely linked to higher-order cognitive processes that may depend on neuroplastic adaptations—such as increased brain-derived neurotrophic factor and changes in endocannabinoid signaling—that are more consistently observed with moderate-to-vigorous activity.^72,73,74,75^ This may suggest that improvements in academic outcomes require higher-intensity physical activity rather than daily movement alone. Academic performance also reflects factors beyond cognitive ability, including domain-specific knowledge and skills, instructional context, and assessment practices.^76,77,78,79^ Therefore, even if daily steps are associated with cognitive function, these associations may be overshadowed by the many noncognitive determinants of academic performance.

We found that greater daily step count was associated with higher cortical thickness in the paracentral and rostral anterior cingulate, which was partially consistent with our hypothesis. The rostral anterior cingulate cortex is involved in response conflict during information processing,^80^ and the paracentral lobule acts as a medial sensorimotor hub for lower-limb motor control and somatosensory processing.^81^ This pattern indicates that daily steps may be linked to structural variation in brain systems spanning both motor and cognitive domains in adolescence. In addition, because cortical thickness typically decreases during normative development,^40,82,83,84^ greater thickness in youth with higher daily step counts may reflect relatively slower cortical thinning, although longitudinal data are needed to determine whether this represents differences in developmental trajectories. Mediation analyses further indicated that greater paracentral thickness associated with higher daily step count was in turn associated with fewer total mental health problems. Although not classically viewed as an affective region, structural abnormalities of the paracentral lobule, including reduced cortical thickness or gray matter volume, have been reported to be associated with vulnerability to poor mental health including psychotic symptoms and conduct disorder,^85,86,87,88^ highlighting the relevance of sensorimotor and attentional systems for psychological functioning. Notably, our mediation findings suggest that the mental health benefits of daily steps may be more associated with sensorimotor mechanisms than to top-down executive control processes during early adolescence. Given the protracted maturation of prefrontal cortex,^89^ benefits of higher physical activity in these regions may emerge later, underscoring the need for longer-term longitudinal studies.

Contrary to our hypothesis, we found no significant association between daily steps and the volume of subcortical regions in the present sample. Previous studies have more consistently linked cardiorespiratory fitness,^90,91,92^ rather than habitual physical activity, to subcortical volume (such as hippocampus and pallidum), suggesting that fitness may be a more relevant indicator for these regions.^18,92^ Furthermore, evidence of physical activity–associated shape differences in subcortical regions suggests that physical activity may induce localized structural changes that are detectable by shape analyses but not by conventional volumetric measures.^14,93^ Moreover, we did not observe any significant associations between daily step count and either total or regional cortical surface area, which contradicts previous findings from nonwalking activity.^13^ Surface area is mainly related to the proliferation of neural progenitor cells and tangential neuronal migration which are largely complete by early childhood and less plastic thereafter than cortical thickness.^83,94^ As a result, the small cumulative benefits of daily steps may be insufficient to induce detectable changes in surface area, which may require higher-intensity, structured activity.

In categorized analysis, each higher step category was associated with fewer mental health problems compared with the under 5000 steps group. These findings diverge from previous studies suggesting a plateau in physical health benefits for children and young-to-middle-aged adults around 8000 steps,^20,95^ and instead support a graded association with mental health benefits continuing to accrue across higher levels of activity.

In contrast, despite a significant linear association between continuous daily step count and regional cortical thickness, categorical analyses across different step levels did not reveal substantial differences. Compared with the lowest level (<5000 steps), only the highest step group (≥12 000 steps) showed significantly greater paracentral thickness; no significant differences were detected across step categories for rostral anterior cingulate. This more limited pattern of categorical findings likely reflects less apparent associations with brain structure, such that only larger contrasts between very low and higher habitual levels are detectable when step count is categorized. One possible explanation is that structural brain maturation is gradual and extends into early adulthood,^96^ which may limit the detectability of differences across activity levels over relatively short observation periods.

### Limitations

While this study has strengths, including device-measured daily steps as a proxy for physical activity, a large, longitudinal, diverse cohort, and a unique mediation design testing the role of the brain in links between step count and mental health, some limitations must be noted. First, daily step counts primarily capture ambulatory activity and may not reflect higher-intensity or multicomponent activities, such as organized sports or unstructured play, which may provide unique psychological and neurodevelopmental benefits.^30^ Second, this is an observational study and causal conclusions cannot be drawn; residual confounding and bidirectional associations remain possible. Future longitudinal studies could use *g*-methods or marginal structural models to better address this issue. Third, selecting candidate mediators in the same sample may have inflated the estimated indirect effects. Effect sizes should therefore be interpreted cautiously. Fourth, we did not differentiate between indoor and outdoor activity. Outdoor environments provide richer and more diverse sensory stimuli, whereas indoor settings tend to be more monotonous, potentially exerting different effects on adolescents’ developmental outcomes.^97,98^ Future research should consider environmental context as a potential moderator. Fifth, mental health and academic outcomes were based on self-reported measures. Future studies using objective academic measures and clinical assessments of mental health are warranted. Sixth, compared with excluded participants, participants in the analytic sample came from more advantaged families and had fewer mental health problems, consistent with previous ABCD studies of step counter and neuroimaging data availability^99,100^; this may limit the generalizability of our findings.

## Conclusions

Our findings in this cohort study demonstrate that a higher daily step count is associated with better mental health 1 year later and regional variation in cortical thickness, highlighting that every step counts toward meaningful benefit. Notably, paracentral thickness was identified as a pathway associating daily steps with mental health. Together, these findings suggest that accumulation of daily steps may be a promising target for promoting adolescent mental health and healthy brain development.

## Notes

Supplement 1. eMethods. eFigure 1. Histograms Showing the Distriution of Age (A), ADI (B), Grades (C), Mental Health (D), INR (E), NDVI (F), Steps (G), and BMI (H) and a Bar Chart Illustrating Parental Education Levels (I) eFigure 2. Correlations Among Key Variables in the Analytic Sample (N=5515) eFigure 3. Directed Acrylic Graph of the Hypothesized Relationships Among Daily Step Count, Brain Structure, and Mental Health and Academic Performance eFigure 4. Participant Flow Diagram eTable 1. Demographic Characteristics of Participants Included in and Excluded from the Analytic Sample eTable 2. Model Output for the Association Between Step Count, Brain Structure, Mental Health, and Academic Performance eTable 3. Mediation Analyses of Daily Step Count and Mental Health via Step-Associated Brain Structure eTable 4. Model Output for the Association Between Daily Step Count and Brain Structure in Participants With Completed 3-Week Step Data eTable 5. Mediation Analyses of Daily Step Count and Mental Health Via Step-Associated Brain Structure in Participants With Complete 3-Week Step Data eTable 6. Sensitivity Analyses of the Associations Between Daily Step Count, Brain Structure, Mental Health, and Academic Performance, Additionally Adjusting for the Medical History Score eTable 7. Mediation Analyses of the Associations Between Daily Step Count, Brain Structure, Mental Health, and Academic Performance, Additionally Adjusting for the Medical History Score eTable 8. Sensitivity Analyses of the Associations Between Daily Step Count, Brain Structure, Mental Health, and Academic Performance, Using BMI z Scores eTable 9. Mediation Analyses of the Associations Between Daily Step Count, Brain Structure, Mental Health, and Academic Performance Through Step-Associated Brain Structure, Using BMI z Scores eTable 10. Propensity Score–Weighted Sensitivity Analyses of the Associations Between Daily Step Count, Brain Structure, Mental Health, and Academic Performance eTable 11. Sensitivity Analyses of the Associations Between Daily Step Count and Brain Structure, Additionally Adjusting for Intracranial Volume eTable 12. Mediation Analyses of the Associations Between Daily Step Count and Mental Health and Academic Performance Through Step-Associated Brain Structure, Additionally Adjusting for Intracranial Volume eTable 13. Sensitivity Analyses of the Associations Between Daily Step Count and Mental Health and Academic Performance, Additionally Adjusting for Prior Mental Health and Academic Performance eTable 14. Sensitivity Analyses of the Indirect Associations Between Daily Step Count and Mental Health and Academic Performance Through Step-Associated Brain Structure, Additionally Adjusting for Prior Mental Health and Academic Performance eTable 15. Sensitivity Analyses of the Associations Between Daily Step Count and Brain Structure, Accounting for Family-Level Clustering Without Site-Level Random Effects eTable 16. Mediation Analyses of the Associations Between Daily Step Count and Mental Health and Academic Performance Through Step-Associated Brain Structure, Accounting for Family-Level Clustering Only eTable 17. Sensitivity Analyses of the Associations Between Daily Step Count and Brain Structure, Additionally Adjusting for Prior Physical Activity eTable 18. Sensitivity Analyses of the Associations Between Daily Step Count and Brain Structure, Additionally Adjusting for Prior Physical Activity eTable 19. Likelihood ratio tests comparing linear and quadratic models eTable 20. Non-linear Model Output for the Association Between Daily Step Count, Mental Health, Academic Performance, and Brain Structure eTable 21. Wear Characteristics of Participants Included in the Analytic Sample

Supplement 2. Data Sharing Statement
