# Supplementary material for: Physical Activity and Adolescent Brain Structure, Mental Health, and Academic Outcomes
Source: JAMA Netw Open. 2026 Oct 1;9(10):e2636973. doi: 10.1001/jamanetworkopen.2026.36973 (PMC13631767; doi:10.1001/jamanetworkopen.2026.36973)
Supplement: Supplementary file 1 [file jamanetwopen-e2636973-s001.pdf]

Supplemental Online Content

Li Q, Paluch A, Rakesh D. Physical Activity, Brain Structure, Mental Health and Academic Performance Among Adolescents. *JAMA Netw Open.* 2026;9(10):e2636973. doi:10.1001/jamanetworkopen.2026.36973

eMethods

eFigure 1. Histograms Showing the Distriution of Age (A), ADI (B), Grades (C), Mental Health (D), INR (E), NDVI (F), Steps (G), and BMI (H) and a Bar Chart Illustrating Parental Education Levels (I)

eFigure 2. Correlations Among Key Variables in the Analytic Sample (N=5515)

eFigure 3. Directed Acrylic Graph of the Hypothesized Relationships Among Daily Step Count, Brain Structure, and Mental Health and Academic Performance

eFigure 4. Participant Flow Diagram

eTable 1. Demographic Characteristics of Participants Included in and Excluded from the Analytic Sample

eTable 2. Model Output for the Association Between Step Count, Brain Structure, Mental Health, and Academic Performance

eTable 3. Mediation Analyses of Daily Step Count and Mental Health via Step-Associated Brain Structure

eTable 4. Model Output for the Association Between Daily Step Count and Brain Structure in Participants With Completed 3-Week Step Data

eTable 5. Mediation Analyses of Daily Step Count and Mental Health Via Step-Associated Brain Structure in Participants With Complete 3-Week Step Data

eTable 6. Sensitivity Analyses of the Associations Between Daily Step Count, Brain Structure, Mental Health, and Academic Performance, Additionally Adjusting for the Medical History Score

eTable 7. Mediation Analyses of the Associations Between Daily Step Count, Brain Structure, Mental Health, and Academic Performance, Additionally Adjusting for the Medical History Score

eTable 8. Sensitivity Analyses of the Associations Between Daily Step Count, Brain Structure, Mental Health, and Academic Performance, Using BMI z Scores

eTable 9. Mediation Analyses of the Associations Between Daily Step Count, Brain Structure, Mental Health, and Academic Performance Through Step-Associated Brain Structure, Using BMI z Scores

eTable 10. Propensity Score–Weighted Sensitivity Analyses of the Associations Between Daily Step Count, Brain Structure, Mental Health, and Academic Performance

eTable 11. Sensitivity Analyses of the Associations Between Daily Step Count and Brain Structure, Additionally Adjusting for Intracranial Volume

eTable 12. Mediation Analyses of the Associations Between Daily Step Count and Mental Health and Academic Performance Through Step-Associated Brain Structure, Additionally Adjusting for Intracranial Volume

eTable 13. Sensitivity Analyses of the Associations Between Daily Step Count and Mental Health and Academic Performance, Additionally Adjusting for Prior Mental Health and Academic Performance

eTable 14. Sensitivity Analyses of the Indirect Associations Between Daily Step Count and Mental Health and Academic Performance Through Step-Associated Brain Structure, Additionally Adjusting for Prior Mental Health and Academic Performance

© 2026 Li Q et al. *JAMA Network Open.*

eTable 15. Sensitivity Analyses of the Associations Between Daily Step Count and Brain Structure, Accounting for Family-Level Clustering Without Site-Level Random Effects

eTable 16. Mediation Analyses of the Associations Between Daily Step Count and Mental Health and Academic Performance Through Step-Associated Brain Structure, Accounting for Family-Level Clustering Only

eTable 17. Sensitivity Analyses of the Associations Between Daily Step Count and Brain Structure, Additionally Adjusting for Prior Physical Activity

eTable 18. Sensitivity Analyses of the Associations Between Daily Step Count and Brain Structure, Additionally Adjusting for Prior Physical Activity

eTable 19. Likelihood ratio tests comparing linear and quadratic models

eTable 20. Non-linear Model Output for the Association Between Daily Step Count, Mental Health, Academic Performance, and Brain Structure

eTable 21. Wear Characteristics of Participants Included in the Analytic Sample

This supplemental material has been provided by the authors to give readers additional information about their work.

© 2026 Li Q et al. *JAMA Network Open.*

Supplemental Methods

Socioeconomic Status (SES)

SES has been associated with physical activity as well as brain, mental, and academic outcomes[1–3], and has been included as a confounder in previous studies[4,5]. Therefore, SES was included as a covariate in the current analyses. Household SES was assessed using the income-to-needs ratio (INR) and highest parental education. INR was calculated as the median value of the income band divided by the federal poverty line corresponding to the household size. Parental education was reported on a scale from 1 to 22 years. We used maximum parent education (recoded in years). Neighborhood SES was measured using the Area Deprivation Index (ADI) at the census tract level. ADI is a national percentile ranking of neighborhood SES, with higher scores reflecting greater disadvantage[6,7].

Body Mass Index (BMI)

Consistent with previous work[5,8], we also covaried for BMI. BMI was calculated using the standard imperial formula: weight (lbs) divided by height (inches) squared, multiplied by 703.

Greenspace Exposure

Given the established association between greenspace exposure and physical activity, and our prior findings of widespread links between greenspace exposure and adolescents' brain, mental, and academic outcomes[9], greenspace exposure was included as a covariate in the present analyses. Residential greenspace within a 1-km radius was estimated via the satellite-based normalized difference vegetation index (NDVI). NDVI was calculated from the difference in Earth's surface reflectance between the visible red (RED) and near-infrared (NIR) portions of the spectrum: NDVI = (NIR - RED) / (NIR + RED). The index was derived from Sentinel-2 satellite data and processed using Google Earth Engine (GEE). All data was collected in 2017, corresponding with the baseline ABCD data collection (1–3). Satellite data of the primary address where participants spend at least 80% of their time was used in analyses.

Acquisition Site

Regarding data collection site, prior ABCD studies have reported substantial scanner-related differences but minimal independent effects of acquisition site on brain measures[10]. When scanner manufacturer and model are included, site explains little additional variance in brain structure[11,12]. In addition, high collinearity between scanner and site may lead to model convergence issues. Therefore, analyses not accounting for site are reported in the Supplementary Results.

FreeSurfer Reconstruction and Parcellation

Cortical surface reconstruction was performed using FreeSurfer. Cortical gray matter and underlying white matter were labeled through nonlinear surface-based registration to an atlas based on individual cortical folding patterns, followed by Bayesian classification. Cortical regions were defined using the Desikan–Killiany atlas, which parcellates each hemisphere into 34 gyral-based regions. Cortical thickness and surface area were calculated at each surface location,

© 2026 Li Q et al. *JAMA Network Open.*

and regional values were obtained by averaging unsmoothed surface-based measures within each cortical parcel. Subcortical structures were labeled using an automated atlas-based volumetric segmentation procedure. Further details of the ABCD structural image processing pipeline have been described previously.[13]

Sensitivity analyses: Prior Physical Activity

Children's participation in extracurricular activities was assessed using the parent-report ABCD Study Sports and Activities Involvement Questionnaire[14]. This questionnaire assessed children's lifetime history of activity involvement, the frequency and duration of their participation, and their activity level over the past year. Weekly physical activity was calculated for each activity by multiplying the number of sessions per week by the duration of each session and then summing across all physical activities to obtain total hours per week.

Sensitivity analyses: Chronic Health

At baseline, caregivers completed a medical history questionnaire adapted from the Missouri Assessment of Genetics Interview for Children Health Services Utilization Questionnaire[15] , covering 18 past-year and lifetime medical conditions. Responses across individual conditions were summed to create a total medical problems score. Participants with scores greater than six were excluded because of concerns about data validity.

Income Bands

1 = Less than $5,000

2 = $5,000 through $8,500

3 = $12,000 through $13,999

4 = $16,000 through $20,500

5 = $25,000 through $30,000

6 = $35,000 through $42,500

7 = $50,000 through $62,500

8 = $75,000 through $87,500

9 = $100,000 through $150,000

10 = $200,000 and greater

List of items contributing to ADI

1. Percent of the block group's population aged ≥ 25 years with < 9 years of education

2. Percent aged ≥ 25 years with greater than or equal to a high school diploma

3. Percent of employed persons ≥16 years of age in white-collar occupations

4. Median family income

5. Income disparity

6. Median home value

7. Median gross rent

8. Median monthly mortgage

9. Percent owner-occupied housing units

10. Percent of civilian labor force population ≥ 16 years of age unemployed

11. Percent of families below the poverty level

12. Percent of population below 150% of the poverty threshold

13. Percent of single-parent households with children < 18 years of age

14. Percent of occupied housing units without a motor vehicle

15. Percent of occupied housing units without a telephone

16. Percent of occupied housing units without complete plumbing

17. Percent of occupied housing units with more than one person per room

List of items contributing to mental health problems in the brief problem monitor

1. I act too young for my age
2. I argue a lot
3. I fail to finish things I start
4. I have trouble concentrating or paying attention
5. I have trouble sitting still
6. I destroy things belonging to others
7. I disobey my parents
8. I disobey at school.
9. I feel worthless or inferior Definition of inferior: less good
10. I act without stopping to think
11. I am too fearful or anxious
12. I feel too guilty
13. I am self-conscious or easily embarrassed
14. I am inattentive or easily distracted
15. I am stubborn
16. I have a hot temper Definition of "hot temper": bad temper, get mad easily
17. I threaten to hurt people
18. I am unhappy, sad, or depressed
19. I worry a lot

© 2026 Li Q et al. *JAMA Network Open.*

Likelihood Ratio Test for Nonlinearity

To examine potential non-linear associations between daily step count and brain and behavioral outcomes, we compared two nested models: a linear model including only [T2step] (equation A1) and a quadratic model additionally including [I(T2step²)] (equation A2). Both models included random intercepts for family ID and site, and were adjusted for age, sex, scanner model, BMI, highest parental education, INR, neighborhood disadvantage, and greenspace exposure. Model fit was assessed using likelihood ratio tests (ANOVA).

A1. Y = Intercept + (1 | family ID) + (1 | site) + β1 (daily step count) + β2 (age) + β3 (sex) + β4 (scanner model) + β5 (BMI) + β6 (highest parental education) + β7 (INR) + β8 (neighborhood disadvantage) + β9 (greenspace exposure) + e.

A2. Y = Intercept + (1 | family ID) + (1 | site) + β1 (daily step count) + β2 (daily step count^2) + β3 (age) + β4 (sex) + β5 (scanner model) + β6 (BMI) + β7 (highest parental education) + β8 (INR) + β9 (neighborhood disadvantage) + β10 (greenspace exposure) +

Supplemental Results

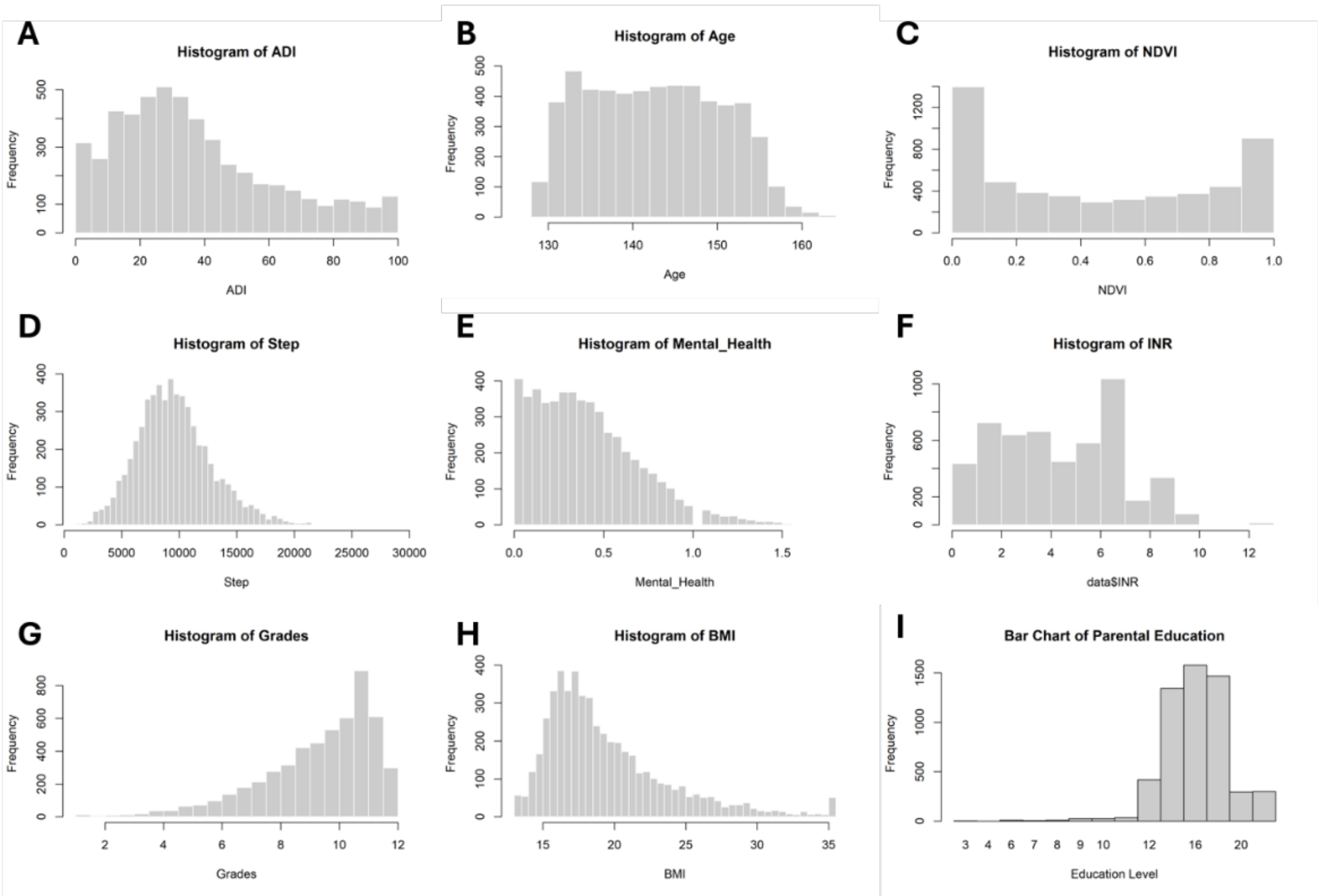

Figure S1. Histograms showing the distribution of ADI (A), age (B), NDVI (C), step (D), mental health (E), INR (F), grades (G), and BMI (H), and a bar chart illustrating parental education levels (I).

© 2026 Li Q et al. *JAMA Network Open.*

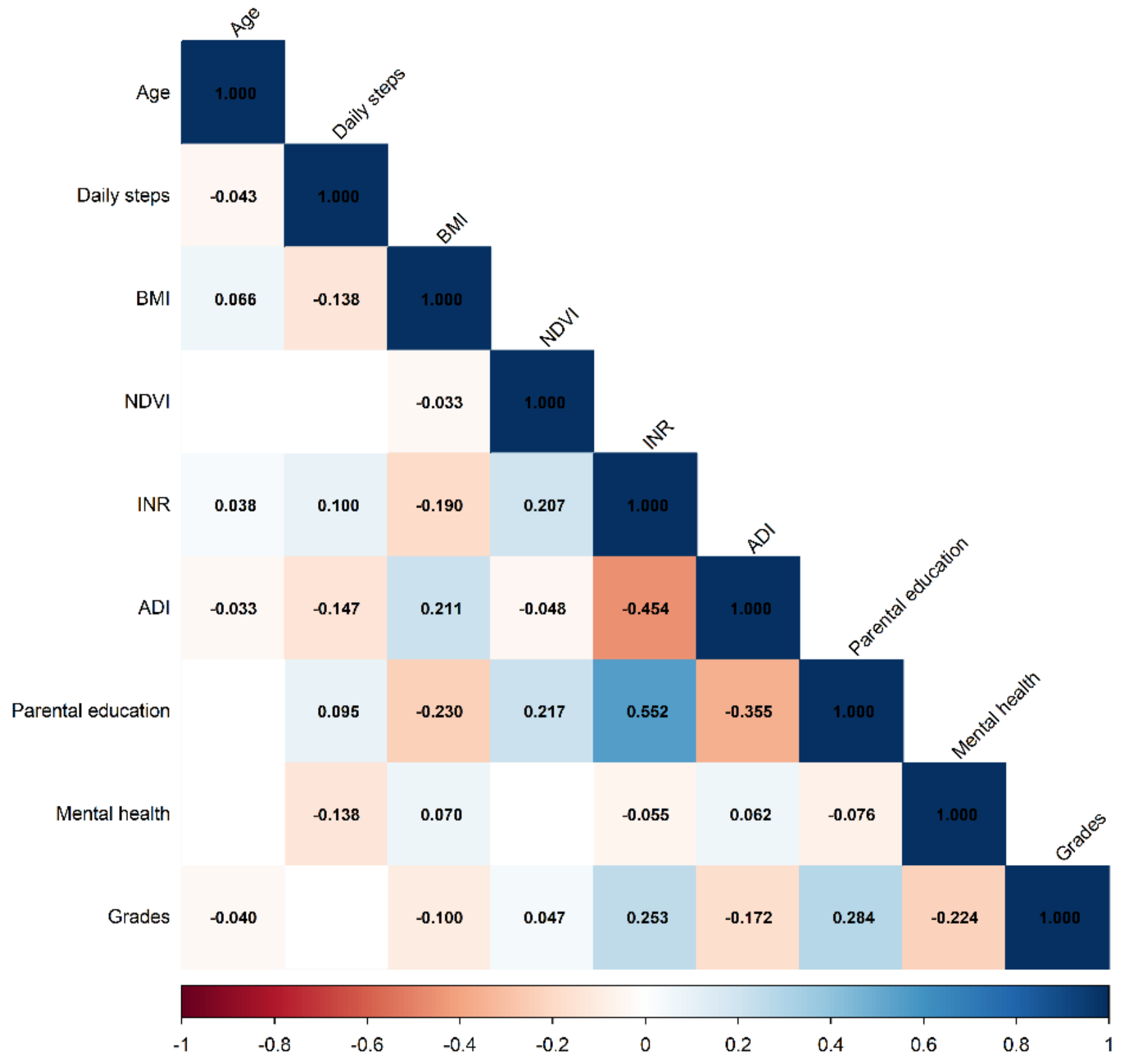

Figure S2. Correlations among key variables in the analytic sample (N=5,515)

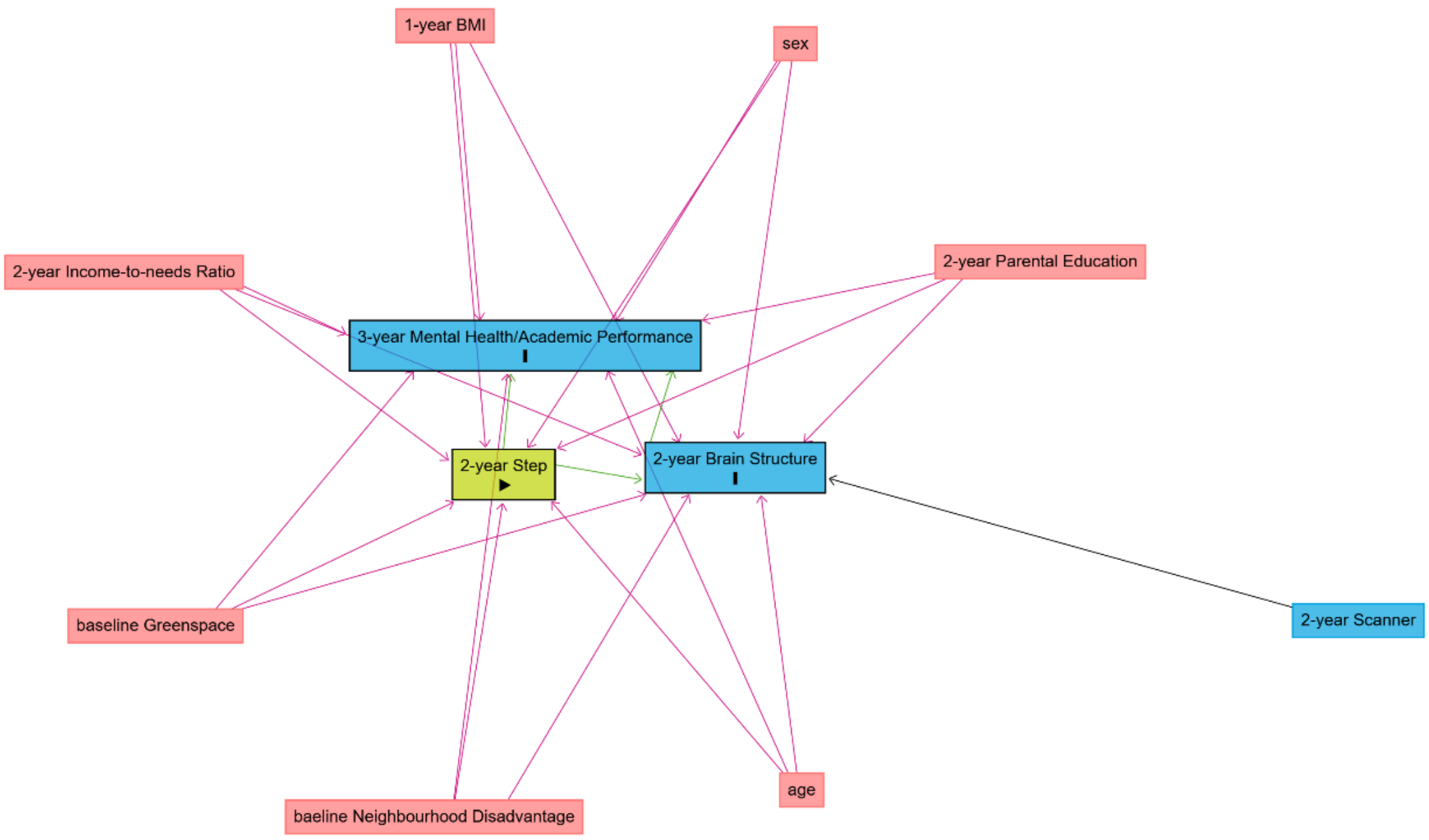

Figure S3. Directed acyclic graph of the hypothesized relationships among daily step count, brain structure, and mental health and academic performance

© 2026 Li Q et al. *JAMA Network Open.*

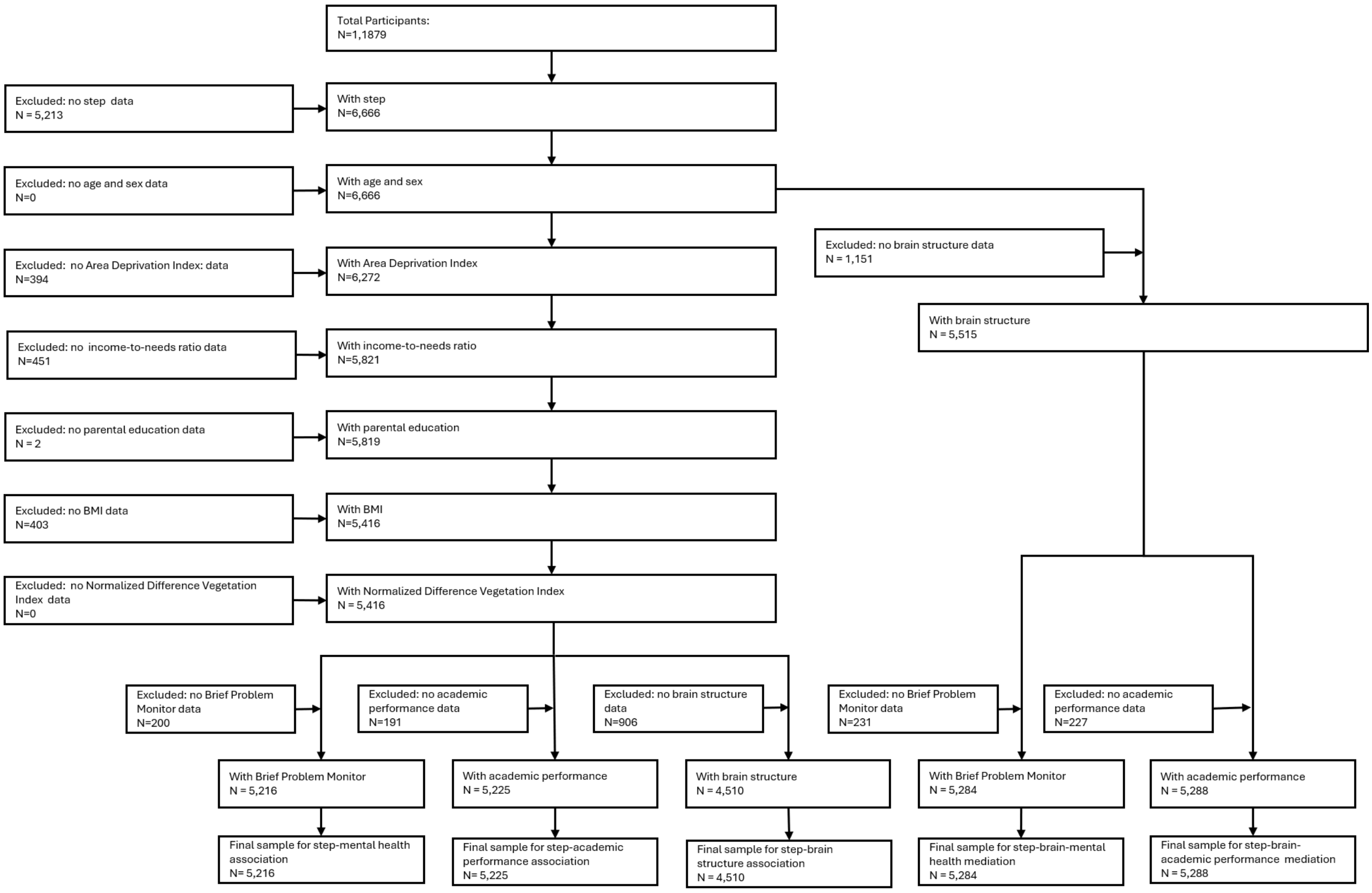

Figure S4. Participant flow diagram

Table S1. Demographic Characteristics of Participants Included in and Excluded from the Analytic Sample

**Characteristic**	**Included: Values (N or Mean ± SD)**	**Excluded: Values (N or Mean ± SD)**
Total Participants (Female Participants)	5,515 (2,640)	6,364 (3,037)
Age, Years	11.93 (± 0.65)	12.13 (± 0.65)
Area Deprivation Index	37.68 (± 24.67)	42.17 (± 28.74)
Income-to-Needs Ratio	4.20 (± 2.39)	3.94 (± 2.63)
Parental Education, Years	16.14 (± 2.65)	15.50 (± 3.03)
Body Mass Index, kg/ m^2	19.44 (± 4.29)	20.05 (± 4.65)
Normalized Difference Vegetation Index	0.46 (± 0.36)	0.42 (± 0.36)

© 2026 Li Q et al. *JAMA Network Open.*

Brief Problem Monitor	0.40 (± 0.30)	0.42 (± 0.32)
Academic Performance	9.54 (± 1.94)	9.10 (± 2.18)

Note. Excluded participants were those without available Fitbit or brain imaging data.

Table S2. Model Output for the Association Between Daily Step Count, Brain Structure, Mental Health, and Academic Performance

**Outcome variable**	***B*(SE)**	***t***	***P* val**
Behavioural outcomes			
Menta health	-0.080(0.015)	-5.497	<.001
Academic performance	0.015(0.014)	0.563	0.573
Whole Brain Measures			
Average cortical thickness	0.032(0.015)	2.16	0.031
Total surface area	-0.027(0.013)	-2.027	0.043
Total cortical volume	-0.009(0.013)	-0.705	0.481
Cortical Thickness			
Banks of the superior temporal sulcus	-0.002(0.016)	-0.11	0.913
Caudal anterior cingulate	0.044(0.016)	2.741	0.006
Caudal middle frontal	0.012(0.016)	0.788	0.43
Cuneus	0.021(0.015)	1.388	0.165
Entorhinal	0.020(0.016)	1.288	0.198
Fusiform	0.013(0.016)	0.86	0.39
Inferior parietal	0.021(0.015)	1.375	0.169
Inferior temporal	0.025(0.016)	1.589	0.112
Isthmus cingulate	0.039(0.016)	2.425	0.015
Lateral occipital	0.007(0.014)	0.536	0.592
Lateral orbitofrontal	0.011(0.015)	0.714	0.475
Lingual	-0.006(0.015)	-0.414	0.679
Medial orbitofrontal	0.030(0.015)	1.975	0.048
Middle temporal	0.028(0.015)	1.873	0.061
Parahippocampal	0.000(0.016)	-0.018	0.986
Paracentral	0.057(0.016)	3.621	<.001
Pars opercularis	0.022(0.015)	1.39	0.165
Pars orbitalis	-0.001(0.016)	-0.083	0.934
Pars triangularis	0.016(0.016)	0.987	0.324
Pericalcarine	0.002(0.015)	0.106	0.916
Postcentral	-0.022(0.015)	-1.435	0.151
Posterior cingulate	0.034(0.016)	2.182	0.029
Precentral	0.017(0.016)	1.108	0.268
Precuneus	0.032(0.016)	2.034	0.042
Rostral anterior cingulate	0.052(0.016)	3.319	0.001
Rostral middle frontal	0.014(0.015)	0.894	0.371
Superior frontal	0.043(0.016)	2.786	0.005
Superior parietal	0.020(0.015)	1.297	0.195
Superior temporal	0.016(0.016)	1.056	0.291
Supramarginal	0.011(0.015)	0.732	0.464

© 2026 Li Q et al. *JAMA Network Open.*

Frontal pole	0.026(0.016)	1.675	0.094
Temporal pole	0.018(0.016)	1.111	0.267
Transverse temporal	-0.020(0.016)	-1.272	0.204
Insula	0.017(0.015)	1.112	0.266
Surface Area			
Banks of the superior temporal sulcus	-0.007(0.015)	-0.48	0.631
Caudal anterior cingulate	-0.034(0.015)	-2.165	0.03
Caudal middle frontal	-0.032(0.015)	-2.078	0.038
Cuneus	0.002(0.015)	0.1	0.92
Entorhinal	-0.011(0.015)	-0.711	0.477
Fusiform	-0.030(0.014)	-2.148	0.032
Inferior parietal	-0.010(0.015)	-0.673	0.501
Inferior temporal	-0.024(0.014)	-1.694	0.09
Isthmus cingulate	-0.030(0.015)	-2.003	0.045
Lateral occipital	-0.006(0.014)	-0.388	0.698
Lateral orbitofrontal	-0.016(0.015)	-1.073	0.283
Lingual	-0.042(0.015)	-2.746	0.006
Medial orbitofrontal	-0.018(0.014)	-1.27	0.204
Middle temporal	-0.022(0.014)	-1.541	0.123
Parahippocampal	-0.018(0.016)	-1.156	0.248
Paracentral	-0.006(0.015)	-0.392	0.695
Pars opercularis	-0.028(0.015)	-1.861	0.063
Pars orbitalis	-0.018(0.014)	-1.235	0.217
Pars triangularis	-0.013(0.015)	-0.872	0.383
Pericalcarine	-0.012(0.016)	-0.78	0.435
Postcentral	-0.022(0.014)	-1.531	0.126
Posterior cingulate	-0.037(0.015)	-2.464	0.014
Precentral	-0.029(0.014)	-2.056	0.04
Precuneus	-0.008(0.015)	-0.564	0.572
Rostral anterior cingulate	-0.021(0.015)	-1.421	0.155
Rostral middle frontal	-0.015(0.014)	-1.068	0.286
Superior frontal	-0.030(0.015)	-2.063	0.039
Superior parietal	-0.018(0.015)	-1.174	0.24
Superior temporal	-0.023(0.015)	-1.545	0.122
Supramarginal	-0.015(0.015)	-0.992	0.321
Frontal pole	-0.031(0.015)	-2.167	0.03
Temporal pole	-0.033(0.015)	-2.217	0.027
Transverse temporal	-0.013(0.016)	-0.806	0.42
Insula	-0.006(0.015)	-0.381	0.703
Subcortical Volume			
Amygdala	-0.022(0.014)	-1.545	0.122
Caudate	-0.020(0.015)	-1.309	0.191
Putamen	-0.008(0.015)	-0.519	0.603
Pallidum	-0.023(0.015)	-1.562	0.118
Hippocampus	-0.037(0.015)	-2.503	0.012
Accumbens area	0.007(0.015)	0.476	0.634
Thalamus proper	-0.022(0.014)	-1.535	0.125

Note. Reported P values are unadjusted for the false discovery rate (FDR).

© 2026 Li Q et al. *JAMA Network Open.*

eTable 3. Mediation Analyses of Daily Step Count and Mental Health and Brain Structure via Step-Associated Brain Structure

Model	Path	*B*	SE	*z*	*P* val
Paracentral Thickness – Mental Health	a	0.053	0.015	3.489	<.001
	b	-0.047	0.014	-3.33	0.001
	c	-0.089	0.015	-6.02	<.001
	Total effect	-0.086	0.015	-5.847	<.001
	Indirect effect	-0.002	0.001	-2.404	0.016
Paracentral Thickness – Grades	a	0.053	0.015	3.456	0.001
	b	0.027	0.014	1.93	0.054
	c	0.019	0.014	1.354	0.176
	Total effect	0.018	0.014	1.254	0.21
	Indirect effect	0.001	0.001	1.701	0.089
Rostral Anterior Cingulate Thickness – Mental Health	a	0.044	0.014	3.054	0.002
	b	0.012	0.014	0.829	0.407
	c	-0.089	0.015	-6.025	<.001
	Total effect	-0.089	0.015	-6.052	<.001
	Indirect effect	0.001	0.001	0.806	0.42
Rostral Anterior Cingulate Thickness – Grades	a	0.045	0.014	3.147	0.002
	b	-0.016	0.013	-1.171	0.242
	c	0.019	0.014	1.357	0.175
	Total effect	0.02	0.014	1.403	0.161
	Indirect effect	-0.001	0.001	-1.099	0.272

Note. Reported *P* values are unadjusted for the false discovery rate (FDR).

eTable 4. Model Output for the Association Between Daily Step Count and Brain Structure in Participants with Complete Three-Week Step Data

Outcome variable	*B*(SE)	*t*	*P* val
Behavioural outcomes			
Menta health	-0.072(0.020)	-3.549	<.001
Academic performance	0.038(0.036)	1.049	0.294
Whole Brain Measures			
Average cortical thickness	0.040(0.020)	2.033	0.042
Total surface area	-0.015(0.017)	-0.874	0.382
Total cortical volume	0.001(0.018)	0.054	0.957
Cortical Thickness			
Banks of the superior temporal sulcus	-0.011(0.020)	-0.527	0.598
Caudal anterior cingulate	0.037(0.020)	1.826	0.068
Caudal middle frontal	0.014(0.020)	0.697	0.486
Cuneus	0.011(0.020)	0.566	0.572
Entorhinal	0.010(0.020)	0.515	0.607
Fusiform	0.039(0.020)	1.933	0.053
Inferior parietal	0.029(0.020)	1.458	0.145
Inferior temporal	0.049(0.020)	2.374	0.018
Isthmus cingulate	0.038(0.021)	1.857	0.063
Lateral occipital	0.022(0.018)	1.257	0.209
Lateral orbitofrontal	0.031(0.020)	1.543	0.123
Lingual	-0.001(0.020)	-0.074	0.941
Medial orbitofrontal	0.043(0.020)	2.196	0.028

© 2026 Li Q et al. *JAMA Network Open.*

Middle temporal	0.033(0.020)	1.691	0.091
Parahippocampal	0.026(0.020)	1.309	0.191
Paracentral	0.080(0.020)	3.981	0
Pars opercularis	0.042(0.020)	2.072	0.038
Pars orbitalis	0.011(0.020)	0.551	0.582
Pars triangularis	0.033(0.020)	1.63	0.103
Pericalcarine	-0.005(0.019)	-0.245	0.807
Postcentral	-0.012(0.020)	-0.626	0.531
Posterior cingulate	0.037(0.020)	1.86	0.063
Precentral	0.031(0.020)	1.534	0.125
Precuneus	0.037(0.020)	1.824	0.068
Rostral anterior cingulate	0.057(0.020)	2.873	0.004
Rostral middle frontal	0.027(0.020)	1.383	0.167
Superior frontal	0.061(0.020)	3.011	0.003
Superior parietal	0.030(0.020)	1.512	0.131
Superior temporal	0.026(0.020)	1.317	0.188
Supramarginal	0.027(0.020)	1.383	0.167
Frontal pole	0.047(0.020)	2.341	0.019
Temporal pole	0.013(0.020)	0.647	0.518
Transverse temporal	0.000(0.020)	0.003	0.997
Insula	0.040(0.020)	2.051	0.04
Surface Area			
Banks of the superior temporal sulcus	-0.017(0.019)	-0.888	0.375
Caudal anterior cingulate	-0.011(0.020)	-0.564	0.573
Caudal middle frontal	-0.023(0.020)	-1.179	0.238
Cuneus	0.005(0.019)	0.28	0.78
Entorhinal	-0.002(0.020)	-0.094	0.925
Fusiform	-0.037(0.018)	-2.066	0.039
Inferior parietal	-0.024(0.019)	-1.266	0.205
Inferior temporal	-0.018(0.018)	-0.988	0.323
Isthmus cingulate	-0.018(0.019)	-0.933	0.351
Lateral occipital	-0.007(0.018)	-0.357	0.721
Lateral orbitofrontal	-0.001(0.019)	-0.044	0.965
Lingual	-0.039(0.020)	-1.984	0.047
Medial orbitofrontal	-0.008(0.018)	-0.451	0.652
Middle temporal	-0.016(0.018)	-0.888	0.375
Parahippocampal	-0.007(0.020)	-0.341	0.733
Paracentral	0.023(0.020)	1.16	0.246
Pars opercularis	-0.029(0.020)	-1.483	0.138
Pars orbitalis	-0.002(0.018)	-0.107	0.915
Pars triangularis	-0.029(0.019)	-1.505	0.132
Pericalcarine	-0.009(0.020)	-0.446	0.656
Postcentral	0.007(0.019)	0.371	0.711
Posterior cingulate	-0.027(0.019)	-1.396	0.163
Precentral	-0.013(0.018)	-0.727	0.468
Precuneus	0.007(0.019)	0.374	0.708
Rostral anterior cingulate	-0.001(0.019)	-0.056	0.955
Rostral middle frontal	-0.006(0.018)	-0.31	0.756
Superior frontal	-0.016(0.019)	-0.88	0.379
Superior parietal	-0.007(0.019)	-0.392	0.695
Superior temporal	-0.012(0.019)	-0.638	0.524
Supramarginal	0.005(0.019)	0.282	0.778
Frontal pole	-0.022(0.018)	-1.183	0.237

© 2026 Li Q et al. *JAMA Network Open.*

Temporal pole	-0.006(0.019)	-0.293	0.77
Transverse temporal	-0.002(0.020)	-0.1	0.92
Insula	0.006(0.019)	0.307	0.759
Subcortical Volume			
Amygdala	-0.020(0.018)	-1.113	0.266
Caudate	-0.015(0.020)	-0.744	0.457
Putamen	0.000(0.019)	0.025	0.98
Pallidum	-0.002(0.019)	-0.095	0.924
Hippocampus	-0.026(0.019)	-1.363	0.173
Accumbens area	0.031(0.019)	1.609	0.108
Thalamus proper	-0.008(0.018)	-0.436	0.663

Note. Reported *P* values are unadjusted for the false discovery rate (FDR).

eTable 5. Mediation Analyses of Daily Step Count and Mental Health via Step-Associated Brain Structure in Participants with Complete Three-Week Step Data

Model	Path	*B*	*SE*	z	*P* val
	a	0.073	0.020	3.737	<.001
	b	-0.046	0.018	-2.581	0.010
Paracentral Thickness – Mental Health	c	-0.080	0.018	-4.372	<.001
	Total effect	-0.076	0.018	-4.172	<.001
	Indirect effect	-0.003	0.002	-2.102	0.036
	a	0.055	0.019	2.937	0.003
	b	0.028	0.018	1.552	0.121
Rostral Anterior Cingulate Thickness – Mental Health	c	-0.080	0.018	-4.374	<.001
	Total effect	-0.081	0.018	-4.453	<.001
	Indirect effect	0.002	0.001	1.386	0.166
	a	0.072	0.020	3.685	<.001
	b	0.041	0.017	2.350	0.019
Paracentral Thickness – Grades	c	0.023	0.018	1.251	0.211
	Total effect	0.020	0.018	1.089	0.276
	Indirect effect	0.003	0.001	1.989	0.047
	a	0.057	0.019	3.049	0.002
	b	-0.013	0.017	-0.772	0.440
Rostral Anterior Cingulate Thickness – Grades	c	0.023	0.018	1.253	0.210
	Total effect	0.024	0.018	1.286	0.199
	Indirect effect	-0.001	0.001	-0.750	0.453

Note. Reported *P* values are unadjusted for the false discovery rate (FDR).

eTable 6. Sensitivity Analyses of the Associations Between Daily Step Count, Brain Structure, Mental Health, and Academic Performance, Additionally Adjusting for Medical History Score

Outcome variable	*B*(SE)	*t*	*P* val
Behavioural outcomes			
Menta health	-0.081(0.016)	-5.082	<.001
Academic performance	0.027(0.030)	0.899	0.368
Whole Brain Measures			
Average cortical thickness	0.024(0.015)	1.588	0.112
Total surface area	-0.027(0.014)	-1.992	0.046
Total cortical volume	-0.014(0.013)	-1.041	0.298
Cortical Thickness			

Banks of the superior temporal sulcus	-0.001(0.016)	-0.064	0.949
Caudal anterior cingulate	0.044(0.016)	2.767	0.006
Caudal middle frontal	0.013(0.016)	0.849	0.396
Cuneus	0.022(0.015)	1.428	0.153
Entorhinal	0.021(0.016)	1.295	0.195
Fusiform	0.012(0.016)	0.782	0.434
Inferior parietal	0.021(0.015)	1.404	0.161
Inferior temporal	0.025(0.016)	1.575	0.115
Isthmus cingulate	0.039(0.016)	2.404	0.016
Lateral occipital	0.007(0.014)	0.526	0.599
Lateral orbitofrontal	0.011(0.016)	0.689	0.491
Lingual	-0.007(0.015)	-0.443	0.658
Middle temporal	0.029(0.015)	1.879	0.06
Parahippocampal	-0.002(0.016)	-0.105	0.916
Paracentral	0.058(0.016)	3.695	<.001
Pars orbitalis	-0.001(0.016)	-0.064	0.949
Pars triangularis	0.018(0.016)	1.124	0.261
Pericalcarine	0.002(0.015)	0.155	0.877
Postcentral	-0.022(0.015)	-1.453	0.146
Posterior cingulate	0.034(0.016)	2.164	0.031
Precentral	0.017(0.016)	1.079	0.281
Precuneus	0.032(0.016)	2.073	0.038
Rostral anterior cingulate	0.053(0.016)	3.381	0.001
Rostral middle frontal	0.014(0.015)	0.945	0.344
Superior frontal	0.044(0.016)	2.838	0.005
Superior parietal	0.020(0.016)	1.273	0.203
Superior temporal	0.017(0.016)	1.084	0.279
Supramarginal	0.012(0.015)	0.763	0.445
Frontal pole	0.028(0.016)	1.77	0.077
Temporal pole	0.018(0.016)	1.142	0.253
Transverse temporal	-0.019(0.016)	-1.217	0.224
Insula	0.018(0.015)	1.147	0.252
Surface Area			
Banks of the superior temporal sulcus	-0.008(0.015)	-0.51	0.61
Caudal anterior cingulate	-0.036(0.016)	-2.3	0.022
Caudal middle frontal	-0.032(0.015)	-2.088	0.037
Cuneus	0.001(0.015)	0.035	0.972
Entorhinal	-0.011(0.015)	-0.727	0.467
Fusiform	-0.031(0.014)	-2.224	0.026
Inferior parietal	-0.012(0.015)	-0.805	0.421
Inferior temporal	-0.026(0.014)	-1.886	0.059
Isthmus cingulate	-0.031(0.015)	-2.057	0.04
Lateral occipital	-0.006(0.014)	-0.406	0.685
Lateral orbitofrontal	-0.019(0.015)	-1.322	0.186
Lingual	-0.041(0.015)	-2.681	0.007
Medial orbitofrontal	-0.021(0.014)	-1.484	0.138
Middle temporal	-0.024(0.014)	-1.697	0.09
Parahippocampal	-0.018(0.016)	-1.173	0.241
Paracentral	-0.008(0.015)	-0.533	0.594
Pars opercularis	-0.029(0.015)	-1.874	0.061
Pars orbitalis	-0.021(0.014)	-1.489	0.137
Pars triangularis	-0.016(0.015)	-1.031	0.303
Pericalcarine	-0.011(0.016)	-0.712	0.477

© 2026 Li Q et al. *JAMA Network Open.*

Postcentral	-0.025(0.014)	-1.725	0.085
Posterior cingulate	-0.040(0.015)	-2.647	0.008
Precentral	-0.031(0.014)	-2.213	0.027
Precuneus	-0.009(0.015)	-0.641	0.522
Rostral anterior cingulate	-0.024(0.015)	-1.574	0.116
Rostral middle frontal	-0.017(0.014)	-1.165	0.244
Superior frontal	-0.033(0.015)	-2.237	0.025
Superior parietal	-0.019(0.015)	-1.269	0.205
Superior temporal	-0.024(0.015)	-1.634	0.102
Supramarginal	-0.017(0.015)	-1.141	0.254
Frontal pole	-0.033(0.015)	-2.276	0.023
Temporal pole	-0.035(0.015)	-2.353	0.019
Transverse temporal	-0.011(0.016)	-0.695	0.487
Insula	-0.008(0.015)	-0.557	0.578
Subcortical Volume			
Amygdala	-0.025(0.014)	-1.778	0.075
Caudate	-0.021(0.015)	-1.368	0.171
Putamen	-0.010(0.015)	-0.647	0.518
Pallidum	-0.026(0.015)	-1.754	0.079
Hippocampus	-0.039(0.015)	-2.642	0.008
Accumbens area	0.003(0.015)	0.233	0.816
Thalamus proper	-0.023(0.014)	-1.6	0.11

Note. Reported *P* values are unadjusted for the false discovery rate (FDR).

eTable 7. Mediation Analyses of the Associations Between Daily Step Count and Mental Health and Academic Performance Through Step-Associated Brain Structure, Additionally Adjusting for Medical History Score

Model	Path	*B*	*SE*	z	*P* val
	a	0.053	0.015	3.496	<.001
	b	-0.047	0.014	-3.34	0.001
Paracentral Thickness – Mental Health	c	-0.085	0.015	-5.757	<.001
	total effect	-0.082	0.015	-5.584	<.001
	Indirect effect	-0.002	0.001	-2.414	0.016
	a	0.045	0.014	3.156	0.002
	b	0.01	0.014	0.746	0.456
Rostral Anterior Cingulate Thickness – Mental Health	c	-0.085	0.015	-5.761	<.001
	total effect	-0.085	0.015	-5.786	<.001
	Indirect effect	0	0.001	0.73	0.465
	a	0.053	0.015	3.455	0.001
	b	0.027	0.014	1.933	0.053
Paracentral Thickness – Grades	c	0.02	0.014	1.435	0.151
	total effect	0.019	0.014	1.335	0.182
	Indirect effect	0.001	0.001	1.7	0.089
	a	0.047	0.014	3.247	0.001
	b	-0.016	0.013	-1.197	0.231
Rostral Anterior Cingulate Thickness – Grades	c	0.02	0.014	1.437	0.151
	total effect	0.021	0.014	1.486	0.137
	Indirect effect	-0.001	0.001	-1.125	0.261

Note. Reported *P* values are unadjusted for the false discovery rate (FDR).

© 2026 Li Q et al. *JAMA Network Open.*

eTable 8. Sensitivity Analyses of the Associations Between Daily Step Count, Brain Structure, Mental Health, and Academic Performance, Using BMI z Scores

**Outcome variable**	***B*(SE)**	***t***	***P* val**
Behavioural outcomes			
Menta health	-0.084(0.016)	-5.299	<.001
Academic performance	0.029(0.029)	0.991	0.322
Whole Brain Measures			
Average cortical thickness	0.035(0.015)	2.328	0.02
Total surface area	-0.025(0.013)	-1.891	0.059
Total cortical volume	-0.007(0.013)	-0.497	0.62
Cortical Thickness			
Banks of the superior temporal sulcus	-0.001(0.016)	-0.044	0.965
Caudal anterior cingulate	0.044(0.016)	2.759	0.006
Caudal middle frontal	0.013(0.016)	0.851	0.395
Cuneus	0.024(0.015)	1.584	0.113
Entorhinal	0.021(0.016)	1.32	0.187
Fusiform	0.016(0.016)	1.044	0.297
Inferior parietal	0.022(0.015)	1.478	0.139
Inferior temporal	0.028(0.016)	1.768	0.077
Isthmus cingulate	0.039(0.016)	2.469	0.014
Lateral occipital	0.010(0.014)	0.758	0.448
Lateral orbitofrontal	0.013(0.015)	0.859	0.391
Lingual	-0.003(0.015)	-0.221	0.825
Medial orbitofrontal	0.032(0.015)	2.091	0.037
Middle temporal	0.031(0.015)	2.055	0.04
Parahippocampal	0.000(0.016)	0.024	0.981
Paracentral	0.059(0.016)	3.789	<.001
Pars opercularis	0.023(0.015)	1.517	0.129
Pars orbitalis	0.000(0.016)	-0.025	0.98
Pars triangularis	0.017(0.016)	1.098	0.272
Pericalcarine	0.002(0.015)	0.156	0.876
Postcentral	-0.021(0.015)	-1.362	0.173
Posterior cingulate	0.035(0.016)	2.248	0.025
Precentral	0.018(0.016)	1.168	0.243
Precuneus	0.033(0.016)	2.142	0.032
Rostral anterior cingulate	0.053(0.016)	3.398	0.001
Rostral middle frontal	0.016(0.015)	1.081	0.28
Superior frontal	0.045(0.016)	2.895	0.004
Superior parietal	0.022(0.015)	1.446	0.148
Superior temporal	0.019(0.016)	1.2	0.23
Supramarginal	0.012(0.015)	0.804	0.421
Frontal pole	0.027(0.016)	1.734	0.083
Temporal pole	0.019(0.016)	1.178	0.239
Transverse temporal	-0.018(0.016)	-1.119	0.263
Insula	0.018(0.015)	1.141	0.254
Surface Area			
Banks of the superior temporal sulcus	-0.006(0.015)	-0.408	0.683
Caudal anterior cingulate	-0.032(0.015)	-2.038	0.042
Caudal middle frontal	-0.031(0.015)	-2.023	0.043
Cuneus	0.002(0.015)	0.131	0.896
Entorhinal	-0.010(0.015)	-0.656	0.512
Fusiform	-0.029(0.014)	-2.091	0.037

© 2026 Li Q et al. *JAMA Network Open.*

Inferior parietal	-0.009(0.015)	-0.587	0.557
Inferior temporal	-0.023(0.014)	-1.643	0.1
Isthmus cingulate	-0.029(0.015)	-1.982	0.048
Lateral occipital	-0.004(0.014)	-0.288	0.773
Lateral orbitofrontal	-0.014(0.015)	-0.961	0.337
Lingual	-0.042(0.015)	-2.723	0.006
Medial orbitofrontal	-0.017(0.014)	-1.18	0.238
Middle temporal	-0.020(0.014)	-1.44	0.15
Parahippocampal	-0.018(0.016)	-1.126	0.26
Paracentral	-0.005(0.015)	-0.314	0.754
Pars opercularis	-0.026(0.015)	-1.695	0.09
Pars orbitalis	-0.016(0.014)	-1.14	0.254
Pars triangularis	-0.012(0.015)	-0.765	0.444
Pericalcarine	-0.012(0.016)	-0.765	0.444
Postcentral	-0.021(0.014)	-1.443	0.149
Posterior cingulate	-0.036(0.015)	-2.381	0.017
Precentral	-0.028(0.014)	-1.993	0.046
Precuneus	-0.006(0.015)	-0.446	0.655
Rostral anterior cingulate	-0.020(0.015)	-1.31	0.19
Rostral middle frontal	-0.014(0.014)	-0.951	0.342
Superior frontal	-0.028(0.015)	-1.943	0.052
Superior parietal	-0.016(0.015)	-1.07	0.285
Superior temporal	-0.021(0.015)	-1.419	0.156
Supramarginal	-0.013(0.015)	-0.888	0.375
Frontal pole	-0.029(0.014)	-2	0.046
Temporal pole	-0.033(0.015)	-2.182	0.029
Transverse temporal	-0.011(0.016)	-0.706	0.48
Insula	-0.004(0.015)	-0.243	0.808
Subcortical Volume			
Amygdala	-0.020(0.014)	-1.45	0.147
Caudate	-0.019(0.015)	-1.236	0.217
Putamen	-0.006(0.015)	-0.437	0.662
Pallidum	-0.022(0.015)	-1.508	0.132
Hippocampus	-0.035(0.015)	-2.371	0.018
Accumbens area	0.008(0.015)	0.519	0.604
Thalamus proper	-0.020(0.014)	-1.429	0.153

Note. Reported *P* values are unadjusted for the false discovery rate (FDR).

eTable S9. Mediation Analyses of the Associations Between Daily Step Count and Mental Health and Academic Performance Through Step-Associated Brain Structure, Using BMI z Scores

Model	Path	*B*	*SE*	*z*	*P* val
Paracentral Thickness - Mental Health	a	0.056	0.015	3.66	<.001
	b	-0.047	0.014	-3.341	0.001
	c	-0.089	0.015	-6.016	<.001
	Total effect	-0.086	0.015	-5.833	<.001
	Indirect effect	-0.003	0.001	-2.462	0.014
Rostral Anterior Cingulate Thickness - Mental Health	a	0.045	0.014	3.161	0.002
	b	0.011	0.014	0.788	0.431
	c	-0.089	0.015	-6.021	<.001
	Total effect	-0.089	0.015	-6.046	<.001
	Indirect effect	0	0.001	0.769	0.442

© 2026 Li Q et al. *JAMA Network Open.*

	a	0.055	0.015	3.628	<.001
	b	0.027	0.014	1.959	0.05
Paracentral Thickness - Grades	c	0.02	0.014	1.376	0.169
	Total effect	0.018	0.014	1.27	0.204
	Indirect effect	0.001	0.001	1.741	0.082
	a	0.046	0.014	3.253	0.001
	b	-0.015	0.013	-1.147	0.251
Rostral Anterior Cingulate Thickness - Grades	c	0.02	0.014	1.38	0.168
	Total effect	0.02	0.014	1.427	0.154
	Indirect effect	-0.001	0.001	-1.083	0.279

Note. Reported *P* values are unadjusted for the false discovery rate (FDR).

eTable S10. Propensity Score–Weighted Sensitivity Analyses of the Associations Between Daily Step Count, Brain Structure, Mental Health, and Academic Performance

Outcome variable	*B*(SE)	*t*	*P* val
Behavioural outcomes			
Menta health	-0.032(0.005)	-6.157	<.001
Academic performance	0.039(0.035)	1.096	0.273
Whole Brain Measures			
Average cortical thickness	0.021(0.018)	1.154	0.249
Total surface area	-0.029(0.016)	-1.859	0.063
Total cortical volume	-0.018(0.016)	-1.102	0.271
Cortical Thickness			
Banks of the superior temporal sulcus	-0.008(0.018)	-0.455	0.649
Caudal anterior cingulate	0.048(0.019)	2.564	0.01
Caudal middle frontal	0.003(0.019)	0.153	0.878
Cuneus	0.018(0.018)	1.034	0.301
Entorhinal	0.028(0.018)	1.563	0.118
Fusiform	0.010(0.018)	0.543	0.587
Inferior parietal	0.023(0.018)	1.268	0.205
Inferior temporal	0.017(0.018)	0.911	0.362
Isthmus cingulate	0.031(0.018)	1.684	0.092
Lateral occipital	0.009(0.016)	0.522	0.601
Lateral orbitofrontal	0.004(0.018)	0.246	0.805
Lingual	0.003(0.018)	0.158	0.874
Medial orbitofrontal	0.020(0.018)	1.123	0.261
Middle temporal	0.017(0.017)	0.989	0.323
Parahippocampal	0.010(0.019)	0.545	0.586
Paracentral	0.057(0.018)	3.121	0.002
Pars opercularis	0.018(0.018)	1.006	0.315
Pars orbitalis	-0.014(0.018)	-0.76	0.447
Pars triangularis	0.007(0.018)	0.419	0.675
Pericalcarine	0.002(0.018)	0.111	0.911
Postcentral	-0.014(0.018)	-0.786	0.432
Posterior cingulate	0.034(0.018)	1.884	0.06
Precentral	0.014(0.018)	0.775	0.438
Precuneus	0.040(0.018)	2.211	0.027
Rostral anterior cingulate	0.039(0.018)	2.196	0.028
Rostral middle frontal	0.006(0.018)	0.346	0.729
Superior frontal	0.042(0.018)	2.322	0.02
Superior parietal	0.028(0.018)	1.501	0.133

Superior temporal	0.011(0.018)	0.581	0.561
Supramarginal	0.014(0.018)	0.815	0.415
Frontal pole	0.021(0.018)	1.196	0.232
Temporal pole	0.011(0.017)	0.616	0.538
Transverse temporal	-0.019(0.018)	-1.038	0.299
Insula	0.012(0.017)	0.69	0.49
Surface Area			
Banks of the superior temporal sulcus	-0.005(0.016)	-0.284	0.776
Caudal anterior cingulate	-0.027(0.018)	-1.547	0.122
Caudal middle frontal	-0.037(0.018)	-2.103	0.036
Cuneus	-0.004(0.019)	-0.191	0.849
Entorhinal	-0.023(0.017)	-1.308	0.191
Fusiform	-0.041(0.016)	-2.537	0.011
Inferior parietal	-0.008(0.017)	-0.49	0.624
Inferior temporal	-0.029(0.016)	-1.821	0.069
Isthmus cingulate	-0.029(0.016)	-1.773	0.076
Lateral occipital	-0.006(0.017)	-0.379	0.705
Lateral orbitofrontal	-0.018(0.017)	-1.05	0.294
Lingual	-0.035(0.017)	-1.979	0.048
Medial orbitofrontal	-0.028(0.016)	-1.679	0.093
Middle temporal	-0.024(0.016)	-1.436	0.151
Parahippocampal	-0.015(0.018)	-0.79	0.43
Paracentral	-0.014(0.018)	-0.81	0.418
Pars opercularis	-0.037(0.017)	-2.102	0.036
Pars orbitalis	-0.020(0.017)	-1.21	0.226
Pars triangularis	-0.025(0.017)	-1.437	0.151
Pericalcarine	-0.012(0.019)	-0.639	0.523
Postcentral	-0.025(0.016)	-1.521	0.128
Posterior cingulate	-0.033(0.017)	-1.908	0.057
Precentral	-0.029(0.016)	-1.815	0.07
Precuneus	-0.012(0.017)	-0.711	0.477
Rostral anterior cingulate	-0.013(0.017)	-0.755	0.451
Rostral middle frontal	-0.012(0.017)	-0.733	0.464
Superior frontal	-0.031(0.017)	-1.846	0.065
Superior parietal	-0.014(0.017)	-0.823	0.411
Superior temporal	-0.028(0.017)	-1.67	0.095
Supramarginal	-0.023(0.017)	-1.376	0.169
Frontal pole	-0.025(0.016)	-1.498	0.134
Temporal pole	-0.044(0.017)	-2.644	0.008
Transverse temporal	-0.011(0.018)	-0.628	0.53
Insula	-0.013(0.017)	-0.727	0.468
Subcortical Volume			
Amygdala	-0.021(0.016)	-1.313	0.189
Caudate	-0.042(0.019)	-2.253	0.024
Putamen	-0.014(0.017)	-0.824	0.41
Pallidum	-0.030(0.017)	-1.738	0.082
Hippocampus	-0.035(0.017)	-2.004	0.045
Accumbens area	0.008(0.018)	0.467	0.64
Thalamus proper	-0.031(0.017)	-1.859	0.063

Note. Reported *P* values are unadjusted for the false discovery rate (FDR).

© 2026 Li Q et al. *JAMA Network Open.*

eTable 11. Sensitivity Analyses of the Associations Between Daily Step Count and Brain Structure, Additionally Adjusting for Intracranial Volume

Outcome variable	*B*(SE)	*t*	*P* val
Whole Brain Measures			
Average cortical thickness	0.029(0.015)	1.886	0.059
Total surface area	0.011(0.008)	1.36	0.174
Total cortical volume	0.024(0.008)	3.193	0.001
Cortical Thickness			
Banks of the superior temporal sulcus	0.001(0.016)	0.052	0.958
Caudal anterior cingulate	0.042(0.016)	2.652	0.008
Caudal middle frontal	0.015(0.016)	0.979	0.328
Cuneus	0.027(0.015)	1.782	0.075
Entorhinal	0.021(0.016)	1.325	0.185
Fusiform	0.018(0.016)	1.184	0.237
Inferior parietal	0.022(0.015)	1.475	0.14
Inferior temporal	0.031(0.016)	1.957	0.05
Isthmus cingulate	0.040(0.016)	2.512	0.012
Lateral occipital	0.014(0.013)	1.058	0.29
Lateral orbitofrontal	0.009(0.015)	0.582	0.56
Lingual	0.004(0.015)	0.244	0.807
Medial orbitofrontal	0.026(0.015)	1.725	0.085
Middle temporal	0.030(0.015)	1.995	0.046
Parahippocampal	-0.001(0.016)	-0.041	0.967
Paracentral	0.060(0.016)	3.871	<.001
Pars opercularis	0.024(0.015)	1.526	0.127
Pars orbitalis	-0.005(0.016)	-0.299	0.765
Pars triangularis	0.015(0.016)	0.945	0.345
Pericalcarine	0.009(0.015)	0.571	0.568
Postcentral	-0.015(0.015)	-0.996	0.319
Posterior cingulate	0.039(0.016)	2.507	0.012
Precentral	0.023(0.015)	1.499	0.134
Precuneus	0.034(0.016)	2.191	0.028
Rostral anterior cingulate	0.049(0.016)	3.134	0.002
Rostral middle frontal	0.012(0.015)	0.774	0.439
Superior frontal	0.039(0.016)	2.518	0.012
Superior parietal	0.023(0.015)	1.512	0.131
Superior temporal	0.020(0.016)	1.301	0.193
Supramarginal	0.014(0.015)	0.917	0.359
Frontal pole	0.022(0.016)	1.397	0.163
Temporal pole	0.014(0.016)	0.862	0.389
Transverse temporal	-0.016(0.016)	-1.04	0.298
Insula	0.017(0.015)	1.126	0.26
Surface Area			
Banks of the superior temporal sulcus	0.019(0.013)	1.421	0.155
Caudal anterior cingulate	-0.006(0.013)	-0.475	0.635
Caudal middle frontal	-0.003(0.013)	-0.208	0.835
Cuneus	0.020(0.014)	1.426	0.154
Entorhinal	0.006(0.015)	0.4	0.689
Fusiform	0.000(0.011)	0.009	0.993
Inferior parietal	0.017(0.012)	1.414	0.158
Inferior temporal	0.004(0.011)	0.386	0.7
Isthmus cingulate	-0.002(0.012)	-0.183	0.855

© 2026 Li Q et al. *JAMA Network Open.*

Lateral occipital	0.019(0.012)	1.534	0.125
Lateral orbitofrontal	0.016(0.011)	1.446	0.148
Lingual	-0.021(0.014)	-1.502	0.133
Medial orbitofrontal	0.015(0.010)	1.406	0.16
Middle temporal	0.008(0.011)	0.694	0.488
Parahippocampal	0.012(0.013)	0.893	0.372
Paracentral	0.021(0.013)	1.622	0.105
Pars opercularis	-0.005(0.014)	-0.364	0.716
Pars orbitalis	0.009(0.012)	0.727	0.467
Pars triangularis	0.009(0.014)	0.63	0.529
Pericalcarine	0.003(0.015)	0.171	0.864
Postcentral	0.007(0.011)	0.65	0.516
Posterior cingulate	-0.007(0.012)	-0.605	0.545
Precentral	-0.002(0.011)	-0.169	0.866
Precuneus	0.022(0.011)	1.969	0.049
Rostral anterior cingulate	0.009(0.012)	0.729	0.466
Rostral middle frontal	0.014(0.012)	1.209	0.227
Superior frontal	0.003(0.011)	0.27	0.787
Superior parietal	0.010(0.013)	0.803	0.422
Superior temporal	0.008(0.011)	0.735	0.462
Supramarginal	0.011(0.012)	0.911	0.362
Frontal pole	-0.005(0.012)	-0.41	0.682
Temporal pole	-0.008(0.013)	-0.577	0.564
Transverse temporal	0.012(0.014)	0.854	0.393
Insula	0.024(0.012)	2.078	0.038
Subcortical Volume			
Amygdala	0.003(0.012)	0.234	0.815
Caudate	0.004(0.013)	0.343	0.732
Putamen	0.014(0.013)	1.083	0.279
Pallidum	0.003(0.012)	0.254	0.8
Hippocampus	-0.008(0.012)	-0.694	0.487
Accumbens area	0.032(0.013)	2.454	0.014
Thalamus proper	0.009(0.010)	0.91	0.363

Note. Reported *P* values are unadjusted for the false discovery rate (FDR).

eTable 12. Mediation Analyses of the Associations Between Daily Step Count and Mental Health and Academic Performance Through Step-Associated Brain Structure, Additionally Adjusting for Intracranial Volume

Model	Path	*B*	*SE*	z	*P* val
	a	0.056	0.015	3.638	<.001
	b	-0.045	0.014	-3.213	0.001
Paracentral Thickness - Mental Health	c	-0.09	0.015	-6.069	<.001
	total effect	-0.087	0.015	-5.892	<.001
	Indirect effect	-0.002	0.001	-2.401	0.016
	a	0.042	0.014	2.927	0.003
	b	0.01	0.014	0.729	0.466
Rostral Anterior Cingulate Thickness - Mental Health	c	-0.09	0.015	-6.074	<.001
	total effect	-0.09	0.015	-6.096	<.001
	Indirect effect	0	0.001	0.712	0.477
	a	0.055	0.015	3.61	<.001
Paracentral Thickness - Grades	b	0.022	0.014	1.609	0.108
	c	0.022	0.014	1.544	0.122

© 2026 Li Q et al. *JAMA Network Open.*

	total effect	0.021	0.014	1.456	0.145
	Indirect effect	0.001	0.001	1.479	0.139
Rostral Anterior Cingulate Thickness - Grades	a	0.043	0.014	3.012	0.003
	b	-0.012	0.013	-0.89	0.373
	c	0.022	0.014	1.547	0.122
	Total effect	0.022	0.014	1.58	0.114
	Indirect effect	-0.001	0.001	-0.855	0.393

Note. Reported P values are unadjusted for the false discovery rate (FDR).

eTable 13. Sensitivity Analyses of the Associations Between Daily Step Count and Mental Health and Academic Performance, Additionally Adjusting for Prior Mental Health and Academic Performance

Outcome Measures	*B*	SE	*t*	df	*P* val
Mental health	-0.035	0.012	-2.871	4805.026	0.004
Academic Performance	0.031	0.02	1.565	4966.933	0.118

Note. Reported P values are unadjusted for the false discovery rate (FDR).

eTable 14. Sensitivity Analyses of the Indirect Associations Between Daily Step Count and Mental Health and Academic Performance Through Step-Associated Brain Structure, Additionally Adjusting for Prior Mental Health and Academic Performance

Model	Path	*B*	*SE*	z	*P* val
Rostral Anterior Cingulate Thickness - Mental Health (adjusting for prior mental health)	a	0.046	0.014	3.174	0.002
	b	0.001	0.012	0.05	0.96
	c	-0.038	0.012	-3.106	0.002
	Total effect	-0.038	0.012	-3.105	0.002
	Indirect effect	0	0.001	0.05	0.96
Rostral Anterior Cingulate Thickness - Grades (adjusting for prior grades)	a	0.045	0.014	3.147	0.002
	b	-0.016	0.01	-1.62	0.105
	c	0.021	0.011	1.94	0.052
	Total effect	0.021	0.011	2.007	0.045
	Indirect effect	-0.001	0.001	-1.437	0.151
Paracentral Thickness - Mental Health (adjusting for prior mental health)	a	0.05	0.015	3.293	0.001
	b	-0.03	0.012	-2.532	0.011
	c	-0.038	0.012	-3.104	0.002
	Total effect	-0.037	0.012	-2.983	0.003
	Indirect effect	-0.001	0.001	-2	0.045
Paracentral Thickness - Grades (adjusting for prior grades)	a	0.053	0.015	3.465	0.001
	b	0.005	0.01	0.464	0.642
	c	0.021	0.011	1.938	0.053
	Total effect	0.02	0.011	1.907	0.057
	Indirect effect	0	0.001	0.463	0.643

Note. Reported P values are unadjusted for the false discovery rate (FDR).

eTable 15. Sensitivity Analyses of the Associations Between Daily Step Count and Brain Structure, Accounting for Family-Level Clustering Without Site-Level Random Effects

Outcome Measures	*B*(SE)	*t*	*P* val
Whole Brain Measures			
Average cortical thickness	0.000(0.000)	2.305	0.021

© 2026 Li Q et al. *JAMA Network Open.*

Total surface area	-0.021(0.014)	-1.586	0.113
Total cortical volume	-0.008(0.013)	-0.605	0.545
Cortical Thickness			
Banks of the superior temporal sulcus	-0.005(0.016)	-0.305	0.76
Caudal anterior cingulate	0.046(0.016)	2.876	0.004
Caudal middle frontal	0.018(0.016)	1.148	0.251
Cuneus	0.026(0.015)	1.669	0.095
Entorhinal	0.022(0.016)	1.389	0.165
Fusiform	0.013(0.016)	0.85	0.395
Inferior parietal	0.022(0.015)	1.445	0.148
Inferior temporal	0.020(0.016)	1.301	0.193
Isthmus cingulate	0.039(0.016)	2.47	0.014
Lateral occipital	0.013(0.014)	0.943	0.346
Lateral orbitofrontal	0.015(0.016)	0.968	0.333
Lingual	-0.004(0.015)	-0.246	0.806
Medial orbitofrontal	0.033(0.015)	2.165	0.03
Middle temporal	0.022(0.015)	1.474	0.141
Parahippocampal	0.001(0.016)	0.073	0.942
Paracentral	0.057(0.016)	3.681	<.001
Pars opercularis	0.018(0.015)	1.194	0.233
Pars orbitalis	-0.001(0.016)	-0.047	0.962
Pars triangularis	0.017(0.016)	1.101	0.271
Pericalcarine	0.005(0.015)	0.353	0.724
Postcentral	-0.023(0.015)	-1.479	0.139
Posterior cingulate	0.035(0.016)	2.262	0.024
Precentral	0.019(0.016)	1.212	0.225
Precuneus	0.034(0.016)	2.204	0.028
Rostral anterior cingulate	0.052(0.016)	3.341	0.001
Rostral middle frontal	0.020(0.015)	1.321	0.187
Superior frontal	0.048(0.016)	3.067	0.002
Superior parietal	0.023(0.016)	1.488	0.137
Superior temporal	0.012(0.016)	0.781	0.435
Supramarginal	0.011(0.015)	0.701	0.483
Frontal pole	0.026(0.016)	1.692	0.091
Temporal pole	0.017(0.016)	1.067	0.286
Transverse temporal	-0.020(0.016)	-1.289	0.197
Insula	0.021(0.016)	1.321	0.187
Surface Area			
Banks of the superior temporal sulcus	-0.007(0.015)	-0.497	0.619
Caudal anterior cingulate	-0.033(0.015)	-2.103	0.036
Caudal middle frontal	-0.029(0.015)	-1.902	0.057
Cuneus	0.003(0.015)	0.207	0.836
Entorhinal	-0.011(0.015)	-0.721	0.471
Fusiform	-0.029(0.014)	-2.061	0.039
Inferior parietal	-0.009(0.015)	-0.63	0.529
Inferior temporal	-0.020(0.014)	-1.467	0.143
Isthmus cingulate	-0.026(0.015)	-1.729	0.084
Lateral occipital	-0.003(0.014)	-0.232	0.817
Lateral orbitofrontal	-0.008(0.015)	-0.537	0.591
Lingual	-0.041(0.015)	-2.69	0.007
Medial orbitofrontal	-0.015(0.014)	-1.04	0.298
Middle temporal	-0.019(0.014)	-1.322	0.186
Parahippocampal	-0.019(0.016)	-1.231	0.218

© 2026 Li Q et al. *JAMA Network Open.*

Paracentral	-0.005(0.015)	-0.333	0.739
Pars opercularis	-0.022(0.015)	-1.45	0.147
Pars orbitalis	-0.012(0.014)	-0.857	0.391
Pars triangularis	-0.009(0.015)	-0.583	0.56
Pericalcarine	-0.010(0.016)	-0.656	0.512
Postcentral	-0.022(0.014)	-1.56	0.119
Posterior cingulate	-0.035(0.015)	-2.322	0.02
Precentral	-0.028(0.014)	-1.959	0.05
Precuneus	-0.004(0.015)	-0.271	0.786
Rostral anterior cingulate	-0.019(0.015)	-1.242	0.214
Rostral middle frontal	-0.012(0.014)	-0.842	0.4
Superior frontal	-0.026(0.015)	-1.815	0.07
Superior parietal	-0.015(0.015)	-0.978	0.328
Superior temporal	-0.021(0.015)	-1.465	0.143
Supramarginal	-0.015(0.015)	-1.008	0.313
Frontal pole	-0.029(0.015)	-1.978	0.048
Temporal pole	-0.032(0.015)	-2.13	0.033
Transverse temporal	-0.012(0.016)	-0.755	0.45
Insula	-0.003(0.015)	-0.191	0.849
Subcortical Volume			
Amygdala	-0.018(0.014)	-1.319	0.187
Caudate	-0.018(0.015)	-1.18	0.238
Putamen	-0.007(0.015)	-0.501	0.617
Pallidum	-0.021(0.015)	-1.456	0.145
Hippocampus	-0.035(0.015)	-2.39	0.017
Accumbens area	0.007(0.015)	0.495	0.621
Thalamus proper	-0.019(0.014)	-1.337	0.181

Note. Reported P values are unadjusted for the false discovery rate (FDR).

eTable 16. Mediation Analyses of the Associations Between Daily Step Count and Mental Health and Academic Performance Through Step-Associated Brain Structure, Accounting for Family-Level Clustering Only

Model	Path	*B*	SE	*z*	*P* val
	a	0.055	0.015	3.658	<.001
	b	-0.050	0.013	-3.918	<.001
Paracentral Thickness - Mental Health	c	-0.093	0.018	-5.252	<.001
	Total effect	-0.091	0.018	-5.070	<.001
	Indirect effect	-0.003	0.001	-4.184	<.001
	a	0.035	0.022	1.608	0.108
	b	0.014	0.013	1.036	0.300
Rostral Anterior Cingulate Thickness - Mental Health	c	-0.094	0.018	-5.258	0.000
	Total effect	-0.094	0.018	-5.281	0.000
	Indirect effect	0.000	0.001	0.895	0.371
	a	0.054	0.015	3.587	0.000
	b	0.028	0.014	1.921	0.055
Paracentral Thickness - Grades	c	0.022	0.014	1.528	0.127
	Total effect	0.020	0.014	1.451	0.147
	Indirect effect	0.002	0.001	1.855	0.064
	a	0.036	0.022	1.626	0.104
Rostral Anterior Cingulate Thickness - Grades	b	-0.012	0.013	-0.902	0.367
	c	0.022	0.014	1.531	0.126
	Total effect	0.022	0.014	1.565	0.118

© 2026 Li Q et al. *JAMA Network Open.*

Indirect effect	0.000	0.001	-0.671	0.502

Note. Reported *P* values are unadjusted for the false discovery rate (FDR).

eTable 17. Sensitivity Analyses of the Associations Between Daily Step Count and Brain Structure, Additionally Adjusting for Prior Physical Activity

**Outcome variables**	***B*(SE)**	***t***	***P* val**
Behavioural outcomes			
Menta health	-0.068(0.015)	-4.542	<.001
Academic performance	0.002(0.027)	0.081	0.936
Whole Brain Measures			
Average cortical thickness	0.023(0.016)	1.474	0.14
Total surface area	-0.025(0.014)	-1.811	0.07
Total cortical volume	-0.013(0.014)	-0.927	0.354
Cortical Thickness	0.000(0.000)		
Banks of the superior temporal sulcus	0.000(0.016)	0.002	0.999
Caudal anterior cingulate	0.040(0.016)	2.456	0.014
Caudal middle frontal	0.007(0.016)	0.407	0.684
Cuneus	0.024(0.016)	1.511	0.131
Entorhinal	0.025(0.016)	1.557	0.12
Fusiform	0.012(0.016)	0.756	0.45
Inferior parietal	0.019(0.016)	1.236	0.216
Inferior temporal	0.023(0.016)	1.456	0.146
Isthmus cingulate	0.039(0.016)	2.415	0.016
Lateral occipital	0.008(0.014)	0.566	0.571
Lateral orbitofrontal	0.015(0.016)	0.958	0.338
Lingual	-0.008(0.015)	-0.509	0.611
Medial orbitofrontal	0.036(0.016)	2.303	0.021
Middle temporal	0.027(0.015)	1.731	0.084
Parahippocampal	-0.003(0.016)	-0.211	0.833
Paracentral	0.049(0.016)	3.075	0.002
Pars opercularis	0.021(0.016)	1.351	0.177
Pars orbitalis	-0.003(0.016)	-0.166	0.868
Pars triangularis	0.013(0.016)	0.808	0.419
Pericalcarine	0.005(0.015)	0.294	0.769
Postcentral	-0.022(0.016)	-1.399	0.162
Posterior cingulate	0.034(0.016)	2.111	0.035
Precentral	0.014(0.016)	0.896	0.371
Precuneus	0.029(0.016)	1.807	0.071
Rostral anterior cingulate	0.052(0.016)	3.235	0.001
Rostral middle frontal	0.016(0.016)	1.051	0.293
Superior frontal	0.043(0.016)	2.726	0.006
Superior parietal	0.016(0.016)	1.033	0.302
Superior temporal	0.012(0.016)	0.783	0.434
Supramarginal	0.010(0.016)	0.613	0.54
Frontal pole	0.030(0.016)	1.888	0.059
Temporal pole	0.018(0.016)	1.093	0.274
Transverse temporal	-0.020(0.016)	-1.231	0.218
Insula	0.020(0.016)	1.248	0.212
Surface Area			
Banks of the superior temporal sulcus	-0.009(0.015)	-0.587	0.557
Caudal anterior cingulate	-0.029(0.016)	-1.828	0.068

© 2026 Li Q et al. *JAMA Network Open.*

Caudal middle frontal	-0.035(0.016)	-2.252	0.024
Cuneus	0.001(0.015)	0.089	0.929
Entorhinal	-0.006(0.016)	-0.395	0.693
Fusiform	-0.031(0.014)	-2.138	0.033
Inferior parietal	-0.011(0.015)	-0.713	0.476
Inferior temporal	-0.023(0.014)	-1.594	0.111
Isthmus cingulate	-0.025(0.015)	-1.621	0.105
Lateral occipital	-0.002(0.015)	-0.105	0.916
Lateral orbitofrontal	-0.016(0.015)	-1.104	0.27
Lingual	-0.044(0.016)	-2.786	0.005
Medial orbitofrontal	-0.022(0.015)	-1.49	0.136
Middle temporal	-0.022(0.014)	-1.523	0.128
Parahippocampal	-0.015(0.016)	-0.944	0.345
Paracentral	-0.010(0.016)	-0.63	0.529
Pars opercularis	-0.029(0.016)	-1.841	0.066
Pars orbitalis	-0.017(0.015)	-1.13	0.258
Pars triangularis	-0.009(0.015)	-0.572	0.567
Pericalcarine	-0.014(0.016)	-0.871	0.384
Postcentral	-0.024(0.015)	-1.651	0.099
Posterior cingulate	-0.037(0.015)	-2.372	0.018
Precentral	-0.030(0.014)	-2.114	0.035
Precuneus	-0.007(0.015)	-0.485	0.628
Rostral anterior cingulate	-0.021(0.015)	-1.337	0.181
Rostral middle frontal	-0.015(0.015)	-1.007	0.314
Superior frontal	-0.032(0.015)	-2.182	0.029
Superior parietal	-0.018(0.015)	-1.183	0.237
Superior temporal	-0.024(0.015)	-1.583	0.113
Supramarginal	-0.016(0.015)	-1.096	0.273
Frontal pole	-0.035(0.015)	-2.386	0.017
Temporal pole	-0.028(0.015)	-1.832	0.067
Transverse temporal	-0.014(0.016)	-0.879	0.379
Insula	-0.011(0.015)	-0.708	0.479
Subcortical Volume			
Amygdala	-0.019(0.014)	-1.321	0.186
Caudate	-0.025(0.016)	-1.632	0.103
Putamen	-0.020(0.015)	-1.354	0.176
Pallidum	-0.034(0.015)	-2.295	0.022
Hippocampus	-0.042(0.015)	-2.798	0.005
Accumbens area	0.002(0.015)	0.16	0.873
Thalamus proper	-0.027(0.015)	-1.862	0.063

Note. Reported P values are unadjusted for the false discovery rate (FDR).

eTable 18. Sensitivity Analyses of the Associations Between Daily Step Count and Brain Structure, Additionally Adjusting for Prior Physical Activity

**Model**	**Path**	***B***	**SE**	***z***	***P* val**
	a	0.048	0.016	3.044	0.002
	b	-0.042	0.014	-3.016	0.003
Paracentral Thickness - Mental Health	c	-0.078	0.014	-5.395	<.001
	Total effect	-0.076	0.014	-5.258	<.001
	Indirect effect	-0.002	0.001	-2.118	0.034
Rostral Anterior Cingulate Thickness - Mental Health	a	0.041	0.015	2.774	0.006

© 2026 Li Q et al. *JAMA Network Open.*

	b	0.013	0.014	0.958	0.338
	c	-0.078	0.014	-5.399	<.001
	Total effect	-0.079	0.014	-5.431	<.001
	Indirect effect	0.001	0.001	0.909	0.363
Paracentral Thickness - Grades	a	0.047	0.016	3.001	0.003
	b	0.023	0.014	1.692	0.091
	c	0.011	0.015	0.779	0.436
	Total effect	0.01	0.015	0.703	0.482
	Indirect effect	0.001	0.001	1.481	0.139
Rostral Anterior Cingulate Thickness - Grades	a	0.042	0.015	2.874	0.004
	b	-0.017	0.014	-1.277	0.201
	c	0.011	0.015	0.781	0.435
	Total effect	0.012	0.015	0.829	0.407
	Indirect effect	-0.001	0.001	-1.174	0.24

Note. Reported *P* values are unadjusted for the false discovery rate (FDR).

eTable 19. Likelihood ratio tests comparing linear and quadratic models

To examine potential nonlinear associations, we compared a linear model including only [T2step] with a quadratic model additionally including [I(T2step^2)] using likelihood ratio tests (ANOVA). Both models were adjusted for age, sex, scanner, bmi, INR, ADI, parental education, ndvi, with random intercepts for site and fam_id. The table reports the chi-square statistic and p-value from the likelihood ratio tests; the quadratic term did not significantly improve model fit for any outcome after FDR correction.

**Outcome Variable**	***χ2***	***P***
Behavioural outcomes		
Menta health	1.473	0.225
Academic performance	1.929	0.165
Whole Brain Measures		
Average cortical thickness	0.278	0.598
Total surface area	0.133	0.716
Total cortical volume	0.047	0.829
Cortical Thickness		
Banks of the superior temporal sulcus	0.776	0.378
Caudal anterior cingulate	2.41	0.121
Caudal middle frontal	0.074	0.785
Cuneus	1.313	0.252
Entorhinal	0.849	0.357
Fusiform	1.846	0.174
Inferior parietal	0.087	0.768
Inferior temporal	6.535	0.011
Isthmus cingulate	0.144	0.705
Lateral occipital	3.062	0.08
Lateral orbitofrontal	0.323	0.57
Lingual	0.01	0.922
Medial orbitofrontal	0.481	0.488
Middle temporal	2.162	0.141
Parahippocampal	2.588	0.108
Paracentral	0.016	0.899
Pars opercularis	0.007	0.934
Pars orbitalis	0	0.996

© 2026 Li Q et al. *JAMA Network Open.*

Pars triangularis	1.792	0.181
Pericalcarine	1.801	0.18
Postcentral	2.455	0.117
Posterior cingulate	0.537	0.464
Precentral	1.664	0.197
Precuneus	1.124	0.289
Rostral anterior cingulate	0.434	0.51
Rostral middle frontal	0.303	0.582
Superior frontal	0.059	0.809
Superior parietal	0.149	0.7
Superior temporal	2.314	0.128
Supramarginal	0.041	0.84
Frontal pole	7.86	0.005
Temporal pole	4.274	0.039
Transverse temporal	0.002	0.967
Insula	3.374	0.066
Surface Area		
Banks of the superior temporal sulcus	2.101	0.147
Caudal anterior cingulate	2.078	0.149
Caudal middle frontal	0.5	0.479
Cuneus	3.581	0.058
Entorhinal	0.009	0.925
Fusiform	0.453	0.501
Inferior parietal	2.416	0.12
Inferior temporal	7.409	0.006
Isthmus cingulate	0.466	0.495
Lateral occipital	3.881	0.049
Lateral orbitofrontal	0.002	0.96
Lingual	0.465	0.495
Medial orbitofrontal	0.153	0.696
Middle temporal	5.396	0.02
Parahippocampal	0.222	0.638
Paracentral	1.173	0.279
Pars opercularis	0.051	0.821
Pars orbitalis	0.53	0.466
Pars triangularis	0	0.994
Pericalcarine	0.123	0.726
Postcentral	0.027	0.87
Posterior cingulate	0.875	0.35
Precentral	0.011	0.915
Precuneus	0.163	0.687
Rostral anterior cingulate	0.011	0.916
Rostral middle frontal	0.029	0.865
Superior frontal	0.452	0.501
Superior parietal	0.151	0.698
Superior temporal	0.352	0.553
Supramarginal	1.359	0.244
Frontal pole	2.544	0.111
Temporal pole	0.114	0.735
Transverse temporal	2.522	0.112
Insula	0.026	0.871
Subcortical Volume		

© 2026 Li Q et al. *JAMA Network Open.*

Amygdala	1.232	0.267
Caudate	0.213	0.644
Putamen	0.374	0.541
Pallidum	0.243	0.622
Hippocampus	0.097	0.755
Accumbens area	0.697	0.404
Thalamus proper	0.45	0.502

eTable 20. Non-linear Model Output for the Association Between Daily Step Count, Mental Health, Academic Performance, and Brain Structure.

We included both linear [T2step] and quadratic [I(T2step^2)] terms for steps in the model. The model was adjusted for age, sex, scanner, bmi, INR, ADI, parental education, ndvi, with random intercepts for site and fam_id. The output for the quadratic term [I(T2step^2)] showed no significant association with any outcome after FDR test. Estimates shown are for the quadratic term [I(T2step²)] from models including both linear and quadratic terms.

Outcome	B(SE)	t	P val
Behavioural outcomes			
Menta health	-0.085(0.015)	-5.497	<.001
Academic performance	0.027(0.028)	0.936	0.349
Whole Brain Measures			
Average cortical thickness	0.021(0.016)	1.319	0.187
Total surface area	-0.023(0.014)	-1.607	0.108
Total cortical volume	-0.011(0.014)	-0.75	0.453
Cortical Thickness			
Banks of the superior temporal sulcus	0.009(0.010)	0.877	0.381
Caudal anterior cingulate	-0.016(0.010)	-1.553	0.12
Caudal middle frontal	0.003(0.010)	0.278	0.781
Cuneus	0.011(0.010)	1.145	0.252
Entorhinal	0.009(0.010)	0.926	0.355
Fusiform	0.013(0.010)	1.357	0.175
Inferior parietal	0.003(0.010)	0.297	0.766
Inferior temporal	0.025(0.010)	2.554	0.011
Isthmus cingulate	-0.004(0.010)	-0.373	0.709
Lateral occipital	0.015(0.008)	1.75	0.08
Lateral orbitofrontal	0.006(0.010)	0.568	0.57
Lingual	0.001(0.009)	0.1	0.921
Medial orbitofrontal	0.007(0.010)	0.691	0.489
Middle temporal	0.014(0.009)	1.462	0.144
Parahippocampal	0.016(0.010)	1.611	0.107
Paracentral	0.001(0.010)	0.13	0.897
Pars opercularis	-0.001(0.010)	-0.098	0.922
Pars orbitalis	0.000(0.010)	-0.005	0.996
Pars triangularis	-0.013(0.010)	-1.327	0.185
Pericalcarine	0.013(0.009)	1.34	0.18
Postcentral	-0.015(0.010)	-1.57	0.116
Posterior cingulate	-0.007(0.010)	-0.733	0.464
Precentral	0.013(0.010)	1.297	0.195
Precuneus	-0.010(0.010)	-1.056	0.291
Rostral anterior cingulate	0.006(0.010)	0.659	0.51
Rostral middle frontal	-0.005(0.010)	-0.544	0.586

© 2026 Li Q et al. *JAMA Network Open.*

Superior frontal	0.002(0.010)	0.247	0.805
Superior parietal	-0.004(0.010)	-0.383	0.702
Superior temporal	0.015(0.010)	1.512	0.131
Supramarginal	0.002(0.010)	0.198	0.843
Frontal pole	0.028(0.010)	2.811	0.005
Temporal pole	0.021(0.010)	2.091	0.037
Transverse temporal	0.000(0.010)	-0.045	0.964
Insula	0.018(0.010)	1.836	0.066
Surface Area			
Banks of the superior temporal sulcus	-0.013(0.009)	-1.42	0.156
Caudal anterior cingulate	0.014(0.010)	1.466	0.143
Caudal middle frontal	-0.007(0.010)	-0.693	0.488
Cuneus	-0.018(0.009)	-1.87	0.061
Entorhinal	0.001(0.010)	0.096	0.923
Fusiform	-0.006(0.009)	-0.663	0.507
Inferior parietal	-0.014(0.009)	-1.528	0.126
Inferior temporal	-0.024(0.009)	-2.702	0.007
Isthmus cingulate	0.007(0.009)	0.7	0.484
Lateral occipital	-0.017(0.009)	-1.949	0.051
Lateral orbitofrontal	0.001(0.009)	0.061	0.951
Lingual	0.007(0.010)	0.687	0.492
Medial orbitofrontal	0.004(0.009)	0.407	0.684
Middle temporal	-0.020(0.009)	-2.299	0.022
Parahippocampal	0.005(0.010)	0.48	0.632
Paracentral	-0.010(0.010)	-1.069	0.285
Pars opercularis	-0.002(0.010)	-0.214	0.831
Pars orbitalis	0.007(0.009)	0.739	0.46
Pars triangularis	0.000(0.009)	0.007	0.994
Pericalcarine	-0.003(0.010)	-0.347	0.729
Postcentral	-0.001(0.009)	-0.16	0.873
Posterior cingulate	0.009(0.009)	0.964	0.335
Precentral	0.001(0.009)	0.113	0.91
Precuneus	0.004(0.009)	0.422	0.673
Rostral anterior cingulate	0.001(0.009)	0.125	0.901
Rostral middle frontal	0.002(0.009)	0.185	0.853
Superior frontal	-0.006(0.009)	-0.657	0.511
Superior parietal	-0.003(0.009)	-0.375	0.708
Superior temporal	0.006(0.009)	0.608	0.543
Supramarginal	0.011(0.009)	1.173	0.241
Frontal pole	-0.014(0.009)	-1.586	0.113
Temporal pole	0.003(0.009)	0.353	0.724
Transverse temporal	0.016(0.010)	1.603	0.109
Insula	-0.001(0.009)	-0.144	0.886
Subcortical Volume			
Amygdala	0.010(0.009)	1.123	0.261
Caudate	0.005(0.009)	0.487	0.626
Putamen	-0.006(0.009)	-0.613	0.54
Pallidum	0.005(0.009)	0.522	0.602
Hippocampus	0.003(0.009)	0.35	0.727
Accumbens area	-0.008(0.009)	-0.842	0.4
Thalamus proper	0.006(0.009)	0.695	0.487

Note. Reported *P* values are unadjusted for the false discovery rate (FDR).

© 2026 Li Q et al. *JAMA Network Open.*

Table S21. Wear Characteristics of Participants Included in the Analytic Sample

Wear characteristics	Mean (±SD)
Wear day	
Weekdays, Days	4.405 (± 0.632)
Weekend, Days	1.696 (± 0.369)
Wear time	
Weekdays, Minutes	885.362 (± 68.870)
Weekend, Minutes	847.000 (± 88.624)
Number of weeks	
three weeks	3,350
two weeks	1,339
one week	826

© 2026 Li Q et al. *JAMA Network Open.*
