# Supplementary material for: Physical Activity and Adolescent Brain Structure, Mental Health, and Academic Outcomes
Source: JAMA Netw Open. 2026 Oct 1;9(10):e2636973. doi: 10.1001/jamanetworkopen.2026.36973 (PMC13631767; doi:10.1001/jamanetworkopen.2026.36973)
Supplement: Supplementary file 2 [file jamanetwopen-e2636973-s002.pdf]

Data Sharing Statement

Li. Physical Activity, Brain Structure, Mental Health, and Academic Performance Among Adolescents. *JAMA Netw Open*. Published October 01, 2026.
doi:10.1001/jamanetworkopen.2026.36973

Data
Data available: No

Additional Information
Explanation for why data not available: The data are publicly available through a DUC process
